# Targeting USP47 overcomes tyrosine kinase inhibitor resistance and eradicates leukemia stem/progenitor cells in chronic myelogenous leukemia

**DOI:** 10.1038/s41467-020-20259-0

**Published:** 2021-01-04

**Authors:** Hu Lei, Han-Zhang Xu, Hui-Zhuang Shan, Meng Liu, Ying Lu, Zhi-Xiao Fang, Jin Jin, Bo Jing, Xin-Hua Xiao, Shen-Meng Gao, Feng-Hou Gao, Li Xia, Li Yang, Li-Gen Liu, Wei-Wei Wang, Chuan-Xu Liu, Yin Tong, Yun-Zhao Wu, Jun-Ke Zheng, Guo-Qiang Chen, Li Zhou, Ying-Li Wu

**Affiliations:** 1 Hongqiao International Institute of Medicine, Shanghai Tongren Hospital/Faculty of Basic Medicine, Department of Pathophysiology, Key Laboratory of Cell Differentiation and Apoptosis of the Chinese Ministry of Education, Shanghai Jiao Tong University School of Medicine, 200025 Shanghai, China; 2 Laboratory of Internal Medicine, The First Affiliated Hospital of Wenzhou Medical University, 325000 Wenzhou, China; 3 Department of Oncology, Shanghai 9th People’s Hospital, Shanghai Jiao Tong University School of Medicine, 639 Zhi Zao Ju Road, 200011 Shanghai, China; 4 Department of Hematology, Xinhua Hospital, Shanghai Jiao Tong University School of Medicine, 200092 Shanghai, China; 5 Department of Hematology, Shanghai First People’s Hospital, Shanghai Jiao Tong University School of Medicine, 200081 Shanghai, China; 6 Department of Pathophysiology, Key Laboratory of Cell Differentiation and Apoptosis of the Chinese Ministry of Education and Chinese Academy of Medical Sciences Research Unit (NO.2019RU043), Shanghai Jiao Tong University School of Medicine, 200025 Shanghai, China; 7 Shanghai Institute of Hematology, State Key Laboratory of Medical Genomics, National Research Center for Translational Medicine at Shanghai, Ruijin Hospital affiliated to Shanghai Jiao Tong University School of Medicine, 200025 Shanghai, China

**Keywords:** Cancer stem cells, Cancer therapeutic resistance, Chronic myeloid leukaemia

## Abstract

Identifying novel drug targets to overcome resistance to tyrosine kinase inhibitors (TKIs) and eradicating leukemia stem/progenitor cells are required for the treatment of chronic myelogenous leukemia (CML). Here, we show that ubiquitin-specific peptidase 47 (USP47) is a potential target to overcome TKI resistance. Functional analysis shows that *USP47* knockdown represses proliferation of CML cells sensitive or resistant to imatinib in vitro and in vivo. The knockout of *Usp47* significantly inhibits BCR-ABL and BCR-ABL^T315I^-induced CML in mice with the reduction of Lin^−^Sca1^+^c-Kit^+^ CML stem/progenitor cells. Mechanistic studies show that stabilizing Y-box binding protein 1 contributes to USP47-mediated DNA damage repair in CML cells. Inhibiting USP47 by P22077 exerts cytotoxicity to CML cells with or without TKI resistance in vitro and in vivo. Moreover, P22077 eliminates leukemia stem/progenitor cells in CML mice. Together, targeting USP47 is a promising strategy to overcome TKI resistance and eradicate leukemia stem/progenitor cells in CML.

## Abstract (web summary)

Resistance to tyrosine kinase inhibitors (TKI) is a limitation to their use in treating chronic myelogenous leukemia (CML). Here, the authors show that targeting the ubiquitin peptidase USP47 overcomes TKI resistance and eliminates leukaemia stem/progenitor cells in primary and xenograft CML murine models.

## Introduction

Chronic myelogenous leukemia (CML) is a hematopoietic stem cell malignancy characterized by the t(9;22)(q34;q11) balanced reciprocal translocation of the Philadelphia (Ph) chromosome, which leads to the generation of *BCR-ABL* oncogenic fusion gene that encodes the chimeric BCR-ABL protein with constitutive kinase activity^1,2^. The introduction of imatinib (IM) in 2001, a tyrosine kinase inhibitor (TKI) that targets BCR-ABL, revolutionized the prognosis of CML^3^. However, TKI resistance, including BCR-ABL-dependent and -independent resistance, is a major problem in IM-based CML treatment. The BCR-ABL-dependent mechanism is mainly mediated through the mutation of the ABL kinase domain, BCR-ABL overexpression, or MDR1 upregulation^4–7^. To overcome IM resistance, the second generation of TKIs, such as dasatinib and nilotinib, has been developed^8^. For the “gatekeeper” mutation T315I, which confers resistance to all first- and second-generation TKIs, the third-generation TKI ponatinib was developed^9^. Nevertheless, the toxicity of ponatinib limits its use in some patients^10^. On the other hand, the underlying mechanisms of BCR-ABL-independent TKI resistance are still not well understood. It has been reported that the leukemia stem cells (LSCs) in CML are insensitive to TKI in a BCR-ABL-independent manner, thereby leading to relapse and minimal residual disease (MRD)^11^. Additionally, the aberrant activation of the PI3K and RAS/MAPK signaling pathways also contributes to BCR-ABL-independent TKI resistance^12–15^. Hence, identifying promising drug targets that overcome TKI resistance via both mechanisms is urgently required to provide new possibilities for CML treatment.

To date, ~100 kinds of deubiquitinating enzymes (DUBs) have been identified^16^. DUBs remove ubiquitin conjugates from their substrates, thereby altering their stabilities, localizations, or activities^17^. Accumulating evidence shows that DUBs are promising targets for cancer treatment, including hematopoietic malignancies. For instance, USP10 is involved in the pathogenesis of FLT3-ITD-positive leukemia^18^; targeting USP1 and USP7 is effective in multiple myeloma cells^19,20^; and USP37 can stabilize PLZF/RARA in acute promyelocytic leukemia^21^. Although USP9X has been demonstrated to be involved in the survival of CML, how DUBs are related to CML pathogenesis is largely unexplored^22,23^.

Ubiquitin-specific peptidase 47 (USP47) is a member of the USP subfamily of DUBs^24^. Similar to other USPs, USP47 regulates cellular activities by removing ubiquitin conjugates from diverse substrates and, thereby, altering their stabilities, localizations, or activities. Specifically, USP47 deubiquitinates and stabilizes MAPK^25^, DNA polymerase β (Polβ)^26^, E-cadherin^27^, β-catenin^28^, SNAIL^29^, YAP^30^, β-Trcp^31^, and katanin-p60^32^. Hence, USP47 is involved in cell proliferation^33^, cell survival^31^, DNA damage repair^26^, NLRP3 inflammasome activation^34^, and epithelial-mesenchymal transition^29^. USP47 plays an important role in cancers such as gastric cancer, medulloblastoma, and colorectal cancer^35–37^; however, its role in CML remains unexplored.

In this study, we reveal the critical role of USP47 in the pathogenesis of CML. Specifically, we demonstrate that USP47 is highly expressed in primary CML cells and promotes cell proliferation, while *Usp47* knockout significantly prolongs the survival of BCR-ABL and BCR-ABL^T315I^-induced CML mice by reducing leukemia stem/progenitor cells. We further demonstrate that USP47 facilitates DNA damage repair by regulating a novel substrate, Y-box binding protein 1 (YB-1). Moreover, we find that P22077, a USP47 inhibitor^38^, substantially eliminates TKI-sensitive cells, TKI-resistant cells, leukemia stem/progenitor cells, and MRD in CML. We propose that USP47 is a promising target to overcome TKI resistance in CML treatment.

## Results

### BCR-ABL regulates USP47 through RAS/ERK and STAT5 pathway in CML

**Fig. 1:**
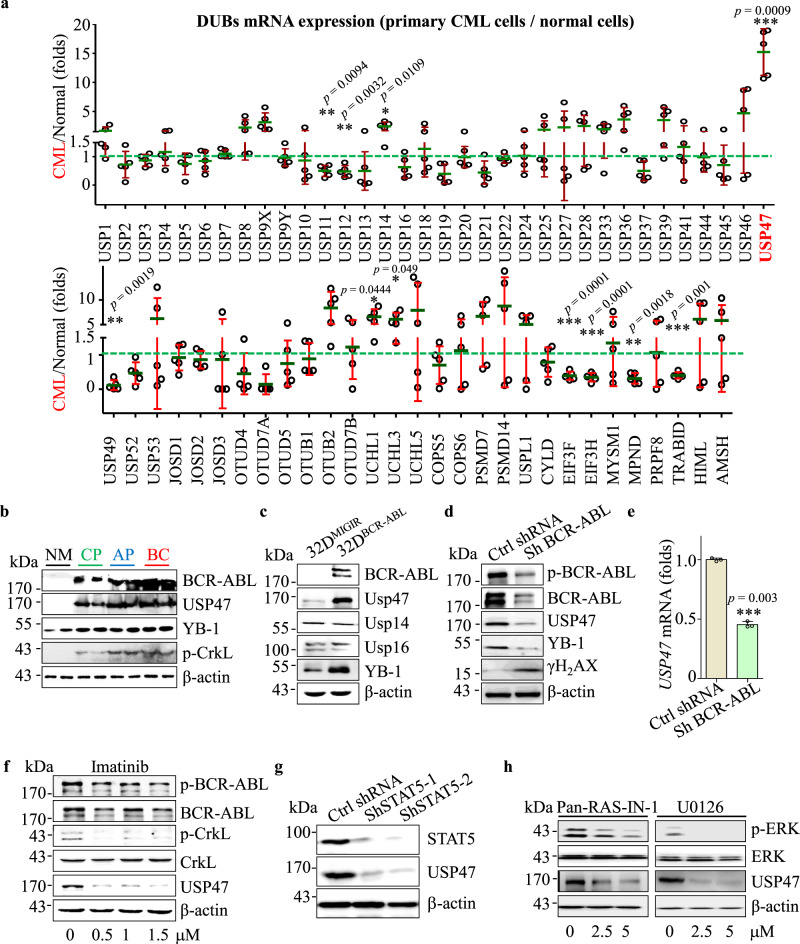
BCR-ABL regulates USP47 through RAS/ERK and STAT5 pathway in CML. **a** mRNA levels of DUBs in bone marrow (BM) mononuclear cells from primary CML patients compared with normal BM CD34^+^ cells (CML *n* = 5, normal *n* = 3 biologically independent samples). Data are presented as mean ± s.d. *p < 0.05; **p < 0.01; ***p < 0.001, using two-sided Student’s *t*-test. **b** USP47 and BCR-ABL protein expression were measured by western blot in primary CML cells at different clinical stages compared with normal BM CD34^+^ cells. NM normal BM, CP chronic phase, AP accelerated phase, BC blast crisis. **c** 32D^MIGIR^ and 32D^BCR−ABL^ cells were collected, and the indicated proteins were examined by western blot. **d**, **e** Protein (**d**) and mRNA (**e**) levels of USP47 in BCR-ABL stably knocked down K562 cells (Sh BCR-ABL) and vector-transfected cells (Ctrl ShRNA) (*n* = 3 biologically independent samples per group). Data are presented as mean ± s.d. ***p < 0.001, using two-sided Student’s *t*-test. **f** K562 cells were treated with IM for 24 h, and the indicated proteins were examined by western blot. **g** *STAT5* knockdown decreased USP47 expression in the K562 cells. **h** Pan-RAS-IN-1 (RAS inhibitor) and U0126 (ERK inhibitor) decreased USP47 expression in the K562 cells after 24 h of treatment. Source data are provided as a Source Data file.

To screen the potential DUBs involved in the pathogenesis of CML, we compared the expression of DUBs in primary CML cells (chronic phase, *n* = 5) and normal bone marrow (BM) CD34^+^ cells (*n* = 3). Compared with normal BM CD34^+^ cells, some of the DUBs including *USP47*, *USP9X*, *USP14*, *OTUB2*, etc. are expressed at higher levels in CML cells, with *USP47* being the most upregulated one (Fig. 1a). Consistent with this result, the USP47 protein level is significantly upregulated in primary CML cells at different stages of disease progression (Fig. 1b). Next, we determined whether USP47 is regulated by BCR-ABL. Compared with the cells in the control group, the expression of *USP47* at the mRNA (Supplementary Fig. 1a) and protein (Fig. 1c) levels are significantly upregulated in BCR-ABL-transfected myeloid progenitor 32D cells (32D^BCR−ABL^). In contrast, BCR-ABL knockdown or IM treatment attenuates the expression of *USP47* at the mRNA and protein levels (Fig. 1d–f). Furthermore, the expression of USP47 is attenuated after RAS or ERK inhibition and STAT5 silence (Fig. 1g, h and Supplementary Fig. 1b, c). These results suggest that BCR-ABL-induced upregulation of USP47 is mediated by the activation of RAS/ERK and STAT5 signaling pathways.

### *USP47* knockdown inhibits proliferation of CML cells

**Fig. 2:**
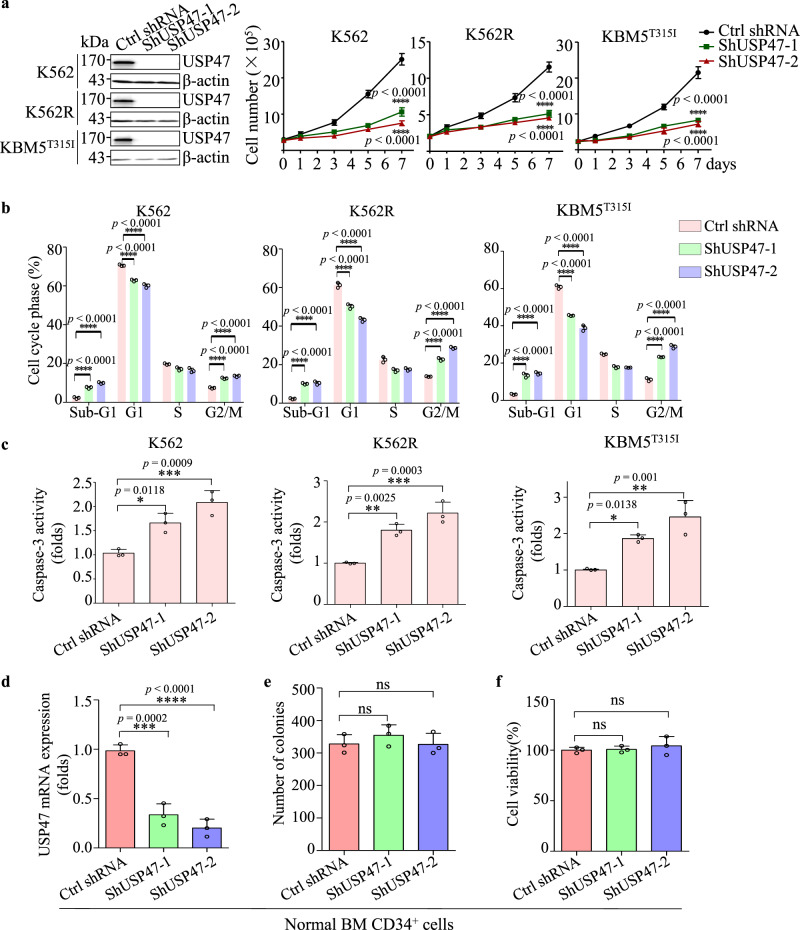
Knockdown of *USP47* inhibits the proliferation of CML cells. **a** *USP47* was silenced in K562, K562R, and KBM5^T315I^ cells using a retroviral transduction system as described in Methods. Viable cells were counted in the transfected cell lines at different times (*n* = 3 biologically independent samples per group). **b** Cell cycle was measured in the transfected cell lines on day 3 (*n* = 3 biologically independent samples per group). **c** Caspase-3 activity was measured after 3 days of retroviral transfection (*n* = 3 biologically independent samples per group). **d** Knockdown efficiency of USP47 in normal human BM CD34^+^ cells was determined by real-time PCR (*n* = 3 biologically independent samples per group). **e**, **f** The number of colonies (**e**) and cell viability (**f**) were measured. Data are mean ± s.d. *p-*values were analyzed by two-way analysis of variance (ANOVA; **a**, **b**) or one-way ANOVA (**c**–**f**). *p < 0.05, **p < 0.01; ***p < 0.001; ****p < 0.0001; ns, no significant. Source data are provided as a Source Data file.

To investigate the role of USP47 in CML, *USP47* was silenced in IM-sensitive K562, IM-resistant K562R, and KBM5^T315I^ cells. As depicted in Fig. 2a, the proliferation of these three cell lines is compromised. Moreover, the numbers of sub-G1 and G2/M phase cells increase after *USP47* knockdown (Fig. 2b). The caspase-3 activity is also increased in *USP47*-knockdown cells (Fig. 2c), indicating the activation of apoptosis in CML cells. Interestingly, *USP47* knockdown in human normal BM CD34^+^ cells does not inhibit cell viability or colony formation ability (Fig. 2d–f). To investigate the function of USP47 in vivo, *USP47*-silenced and control K562 cells were subcutaneously injected into nude mice. Compared with the control group, knockdown of *USP47* remarkably reduces the tumor volumes (Supplementary Fig. 2a–c). Immunohistochemical staining shows that the percentages of PCNA-positive cells are significantly reduced, whereas the percentages of γH_2_AX (a well-recognized DNA damage marker) positive cells are markedly increased compared with the control group (Supplementary Fig. 2d). These data suggest that USP47 is involved in promoting CML cell proliferation both in vitro and in vivo.

### *Usp47* knockout suppresses BCR-ABL-induced CML in mice

**Fig. 3:**
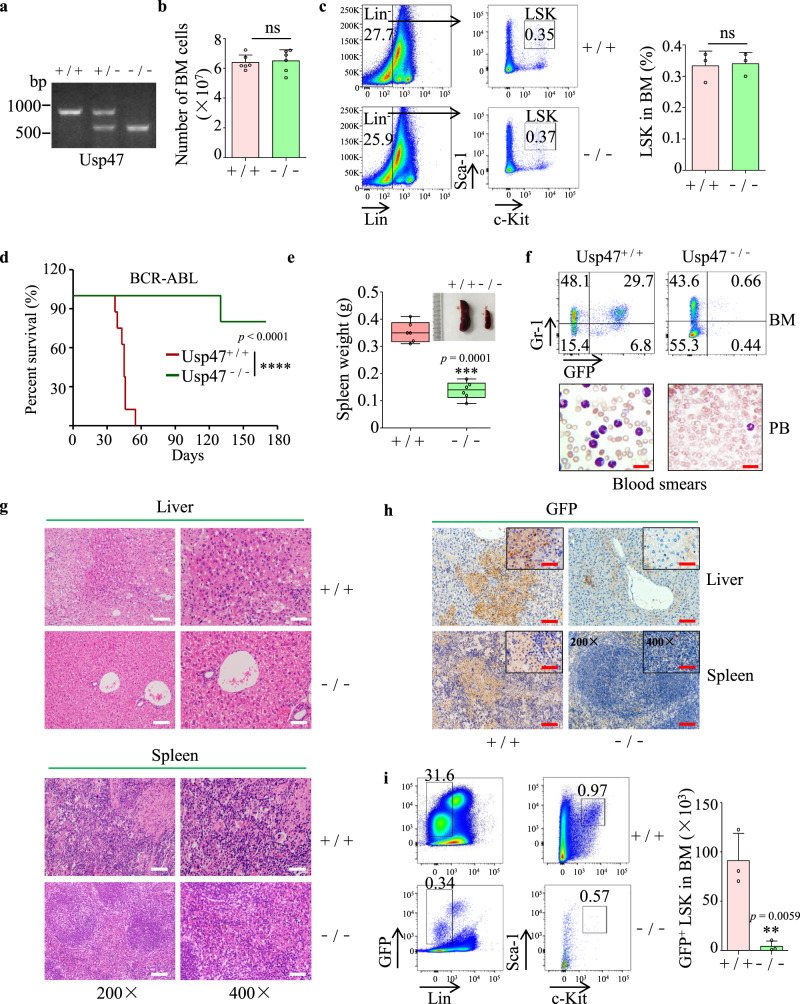
*Usp47* knockout suppresses the development of BCR-ABL-induced CML in mice. **a** Genotyping of *Usp47* knockout (*Usp47*^*−/−*^) mice by PCR. **b** The total BM cells from 8-week-old *Usp47*^*+/+*^ and *Usp47*^*−/−*^ mice were counted (*n* = 6 biologically independent samples per group). Data are mean ± s.d. *p-*values were analyzed by two-sided Student’s *t*-test. ns, no significant. **c** Lin^−^Sca1^+^ c-kit^+^ (LSK) cells from 8-week *Usp47*^*+/+*^ and *Usp47*^*−/−*^ mice (*n* = 3 biologically independent samples per group) were measured by FACS. Data are mean ± s.d. *p-*values were analyzed by two-sided Student’s *t*-test. ns no significant. **d** Survival of mice after receiving BCR-ABL-transduced *Usp47*^*+/+*^ or *Usp47*^*−/−*^ BM cells (*n* = 8 biologically independent samples per group). The experiment was repeated three times. ****p < 0.0001, *p-*value was analyzed by Mantel-Cox-log-rank test. **e** The size and weight of the spleen in the two groups of mice were shown on day 45 (*n* = 6 biologically independent samples per group). center lines of the box and whisker plot represent median values, whereas the box edges indicate the 25th and 75th centiles, and the whiskers indicate the minimum and maximum values. **f** 35 days after transplantation, the percentages of GFP^+^ and Gr-1^+^ cells in BM were examined by FACS analysis (upper panel). The morphology of the cells from PB was examined by Wright-Giemsa staining (lower panel). Scale bar, 20 μm. **g** H&E staining of liver and spleen from the two groups of mice 45 days after transplantation. Scale bars, 200 × , 100 μm; 400 × , 50 μm. **h** Immunohistochemical staining of liver and spleen with GFP antibody 45 days after transplantation. Scale bars, 200 × , 100 μm; 400 × , 50 μm. **i** FACS analysis of the GFP^+^ LSK cells in BM 35 days after transplantation (*n* = 3 biologically independent samples per group). Data are mean ± s.d. *p-*values were analyzed by two-sided Student’s *t*-test. **p < 0.01. Source data are provided as a Source Data file.

To further explore the role of USP47 in CML, the *Usp47* knockout mouse model (*Usp47*^−*/*−^) was established (Fig. 3a). Compared with *Usp47*^*+/+*^ mice, the numbers of total BM cells, total white blood cells, red blood cells, granulocytes, lymphocytes, and platelets are comparable with those in *Usp47*^−*/*−^ mice (Fig. 3b and Supplementary Fig. 3a). The hematopoietic systems of *Usp47*^−*/*−^ and *Usp47*^*+/+*^ mice are also similar (Supplementary Fig. 3b, c). Moreover, the percentages of the bone marrow Lin^−^Sca1^+^c-kit^+^ (LSK) cells, which are the target cells for BCR-ABL, are also comparable between *Usp47*^*+/+*^ and *Usp47*^−*/*−^ mice (Fig. 3c). However, the mice received *BCR-ABL*-transformed *Usp47*^−/−^ BM cells survived significantly longer than those receiving *BCR-ABL*-transformed *Usp47*^*+/+*^ cells (Fig. 3d). Moreover, the size and weight of the spleens in *Usp47*^*+/+*^ mice are significantly increased than those of the *Usp47*^−/−^ mice (Fig. 3e). Flow cytometry analysis showed that the percentage of GFP^+^Gr-1^+^ cells in BM is significantly lower in the *Usp47*^−*/*−^ group compared with that in the *Usp47*^*+/+*^ group (Fig. 3f, upper panel). Consistently, fewer CML leukemia cells were detected in the PB of *Usp47*^−*/*−^ group (Fig. 3f, lower panel). Also, the liver and spleen tissues were more seriously damaged in the *Usp47*^*+/+*^ group than in the *Usp47*^−*/*−^ group (Fig. 3g). In addition, substantial infiltration of CML leukemia cells (GFP-positive cells) was detected in the liver and spleen of the *Usp47*^*+/+*^ mice (Fig. 3h). Furthermore, the number of CML leukemia stem/progenitor cells (GFP^+^ LSK cells) in the *Usp47*^−*/*−^ group is significantly decreased compared with the *Usp47*^*+/+*^ group (Fig. 3i). These data demonstrate that USP47 plays a vital role in the pathogenesis of BCR-ABL-induced CML.

### Silencing *Usp47* inhibits BCR-ABL^T315I^-induced CML and proliferation of KBM^T315I^ in a xenograft mouse model

**Fig. 4:**
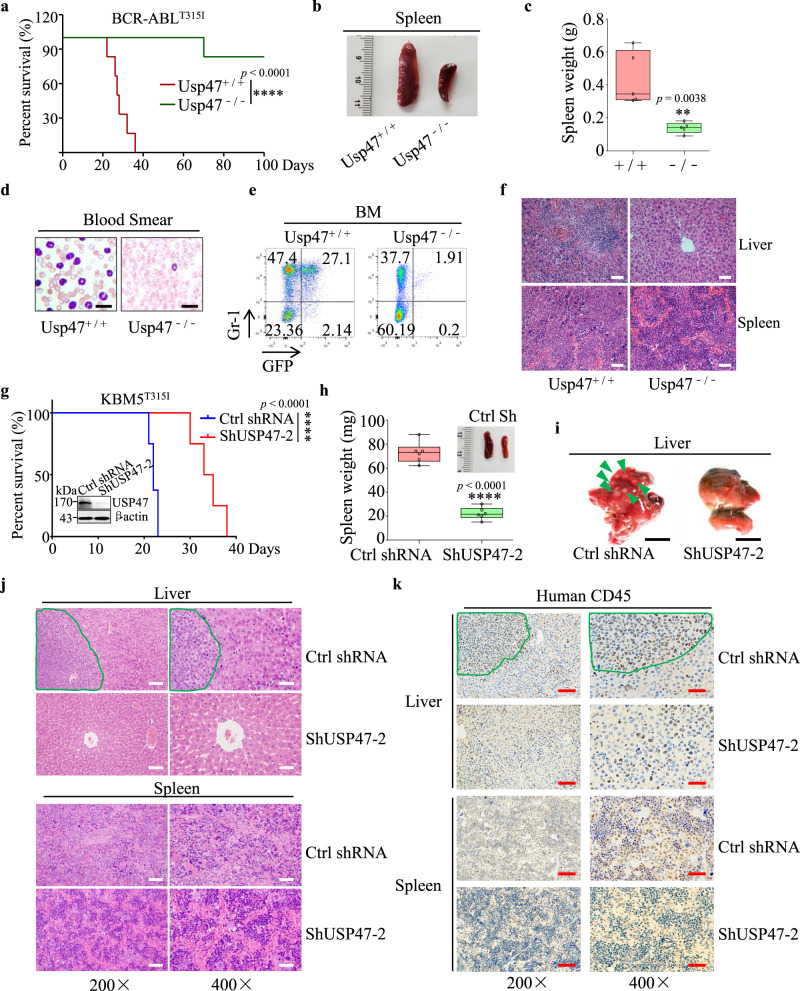
Silence of Usp47 inhibits BCR-ABL^T315I^-induced CML and inhibits proliferation of KBM^T315I^ in a xenograft model. **a** Survival of mice after the transplantation of BCR-ABL^T315I^-transduced *Usp47*^*+/+*^ or *Usp47*^*−/−*^ BM cells (*n* = 6 biologically independent samples per group). ****p < 0.0001, *p*-value was analyzed by Mantel-Cox-log-rank test. The experiment was repeated three times. **b**, **c** Size (**b**) and weight (**c**) of the spleen in the two groups of mice (*n* = 5 biologically independent samples per group) at 28 days after transplantation. Center lines of the box and whisker plot represent median values, whereas the box edges indicate the 25th and 75th centiles, and the whiskers indicate the minimum and maximum values. **d**, **e** At 28 days after transplantation, the morphology of cells from PB (**d**) was examined by Wright-Giemsa staining; the percentages of GFP^+^ and Gr-1^+^ cells (**e**) in BM were examined by FACS analysis. **f** H&E staining of the liver and spleen at 28 days after transplantation. Scale bars, 50 μm. **g** Ctrl shRNA or ShUSP47-2 transfected KBM5^T315I^ cells (*n* = 8 biologically independent samples per group) were injected into B-NDG mice through the tail vein. ****p < 0.0001, *p-*value was analyzed by Mantel-Cox-log-rank test. **h** Size and weight of spleen at day 21 (*n* = 6 biologically independent samples per group). Center lines of the box and whisker plot represent median values, whereas the box edges indicate the 25th and 75th centiles, and the whiskers indicate the minimum and maximum values. **i** Infiltration of leukemia cells in the livers of the mice from Fig. 4h. Green arrows indicate infiltrated leukemic cells. Scale bars, 1 cm. **j** H&E staining of liver and spleen from mice bearing leukemia. Scale bars, 200 × , 100 μm; 400 × , 50 μm. **k** Immunohistochemical staining with human CD45 antibody in the liver and spleen shown in Fig. 4j. Scale bars, 200 × , 100 μm; 400 × , 50 μm. Data are mean ± s.d. *p-*values were analyzed by two-sided Student’s *t*-test (**c**, **h**) or Mantel-Cox-log-rank test (**a**, **g**). **p < 0.01; ****p < 0.0001. Source data are provided as a Source Data file.

Next, we examined the role of Usp47 in a BCR-ABL^T315I^-driven CML mouse model. Consistent with the findings above, *Usp47* depletion substantially prolongs the survival of CML mice compared with the *Usp47*^*+/+*^ group (Fig. 4a). Compared with *Usp47*^*+/+*^ mice, the spleen size and weight in *Usp47*^−*/*−^ mice are significantly decreased (Fig. 4b, c). Besides, there is a remarkable decrease in the number of CML leukemia cells in the PB and BM of the *Usp47*^−*/*−^ mice (Fig. 4d, e). Flow cytometry analysis shows that the number of GFP^+^Gr-1^+^ cells is comparable between the *Usp47*^*+/+*^ and *Usp47*^−*/*−^ mice at 14 days after CML model establishment, while the number of GFP^+^Gr-1^+^ cells decreases in *Usp47*^−*/*−^ mice as time goes by (Supplementary Fig. 4a). The liver and spleen tissues are severely damaged in the *Usp47*^*+/+*^ group compared with the *Usp47*^−*/*−^ mice (Fig. 4f). Additionally, *Usp47* knockout does not affect the homing efficacy of CML LSK cells (Supplementary Fig. 4b). However, the number of CML GFP^+^ LSK cells in the *Usp47*^−*/*−^ group is significantly lower than that in the *Usp47*^*+/+*^ group (Supplementary Fig. 4c). To further validate this finding in human CML cells, we inoculated the *USP47*-knockdown KBM5^T315I^ (ShUSP47-2) and the control KBM5^T315I^ (Ctrl shRNA) cells into B-NDG mice. Compared with the control group, the mice in the ShUSP47-2 group show more prolonged survival with smaller spleen size and weight (Fig. 4g, h). Moreover, leukemic infiltration in the livers of the control mice is more obvious than that of the ShUSP47-2 mice (Fig. 4i). Also, the hematoxylin and eosin (H&E) staining results show that the extent of leukemic infiltration in the liver and spleen in the ShUSP47-2 group is lower than the control group (Fig. 4j). These observations are further confirmed by CD45 antibody staining (Fig. 4k). These data further reveal the vital role of USP47 in the pathogenesis of CML with T315I mutation.

### USP47 interacts with and stabilizes YB-1 protein

**Fig. 5:**
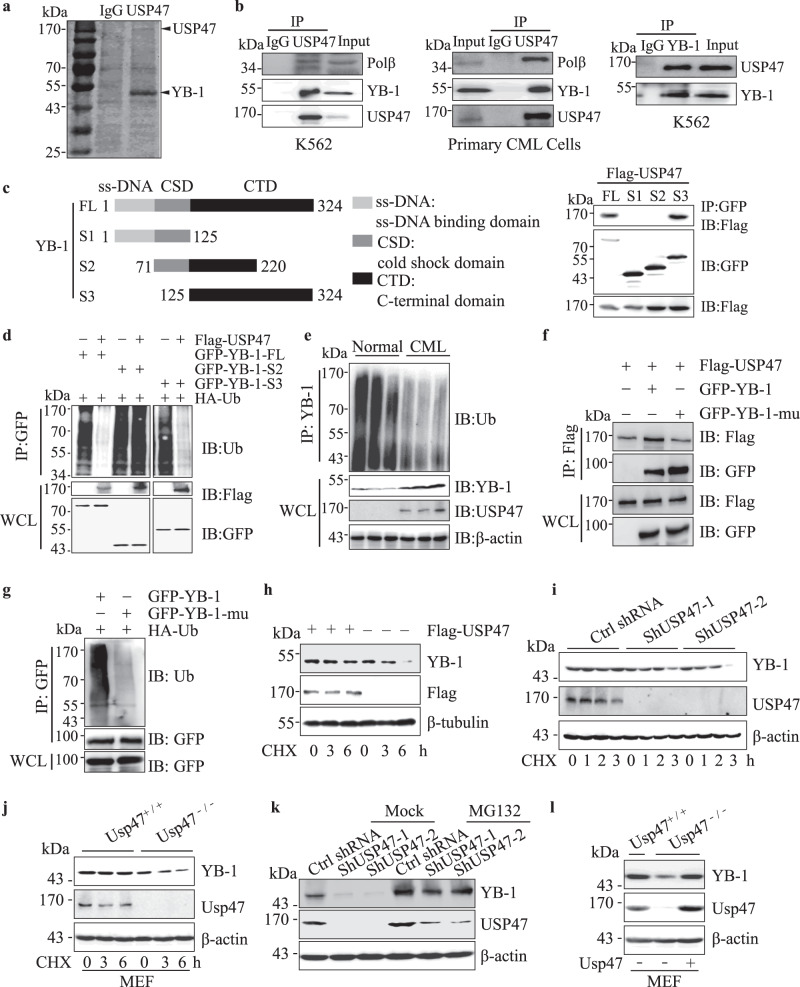
USP47 interacts with YB-1 and protects it from proteasomal degradation. **a** Immunoprecipitation with USP47 antibody in K562 cells, the proteins were separated by SDS-PAGE and visualized by Coomassie brilliant blue (G250). **b** The interaction between endogenous USP47 and YB-1 was analyzed by western blot in K562 and primary CML cells. **c** Full-length or several deletion constructs of GFP-YB-1 was co-transfected with Flag-USP47 in HEK293T cells. Their interactions were examined by co-IP with anti-GFP antibody and by western blot with anti-Flag antibody. **d** GFP-YB-1-FL/GFP-YB-1-S2/GFP-YB-1-S3 and HA-ubiquitin plasmids were co-transfected with or without Flag-USP47 into HEK293T cells. YB-1 was co-IP with anti-GFP antibody, and its ubiquitination was measured with Ub antibody by western blot. **e** Normal and CML BM mononuclear cells were lysed and co-IP with YB-1 antibody, YB-1 ubiquitination was detected with Ub antibody. **f** Flag-USP47, GFP-YB-1, and GFP-YB-1-mu (K137A, K164A, and K170A) were transfected in HEK293T cells. Their interactions were examined by co-IP with anti-Flag antibody and measured by western blot with anti-GFP antibody. **g** GFP-YB-1 or GFP-YB-1-mu was transfected with HA-Ub in HEK293T cells. YB-1 ubiquitination in transiently transfected cells was analyzed by co-IP with anti-GFP antibody and western blot with Ub antibody. **h** USP47 plasmids and empty vectors were transfected into K562 cells. The transfected cells were treated with cycloheximide (CHX, 10 μM) at different times. The indicated proteins were determined by western blot. **i**, **j** *USP47* knockdown cells or *Usp47*^*−/−*^ MEFs were treated with cycloheximide (CHX, 10 μM) at different times together with the control cells, and the indicated proteins were determined by western blot. **k** Control and USP47 stably knockdown K562 cells were treated with vehicle and MG132 (10 μM) for 4 h. The proteins were then extracted and subjected to western blot. **l** YB-1 expression in *Usp47*^*−/−*^ MEFs was measured by western blot after reintroduction of USP47. Source data are provided as a Source Data file.

We explored the mechanism through which USP47 functions in CML. Although it has been reported that MAPK or β-catenin interacts with USP47 in *Drosophila* or tumors^25,28^, USP47 knockdown does not affect the expression of MAPK or β-catenin in CML cells (Supplementary Fig. 5a). We then immunoprecipitated, separated, and analyzed the USP47-interacting proteins using mass spectrometry (Fig. 5a). It was identified that YB-1 can interact with USP47 in K562 and primary CML cells (Fig. 5b). To map the interaction domain of YB-1 with USP47, truncated forms of YB-1 (GFP tag) were constructed and co-transfected with USP47 (Flag tag) into HEK293T cells. The results show that USP47 interacts with the C-terminal domain (CTD) domain of YB-1 (Fig. 5c). We further examined if USP47 regulates the ubiquitination of YB-1. As shown in Fig. 5d, USP47 overexpression significantly inhibits ubiquitination of full-length YB-1 and YB-1-S3, but not that of the truncated YB-1-S2, which cannot bind USP47. Consistent with the deubiquitinating effect of USP47 on YB-1, less ubiquitinated YB-1 is observed in primary CML cells than that in normal BM cells (Fig. 5e). Interestingly, the interaction between YB-1 and USP47 is not affected by the ubiquitination status of YB-1, as the mutation of lysine residues (Lys137, −164, and −170) abrogates the ubiquitination of YB-1, but not its interaction with USP47 (Fig. 5f, g). We then investigated whether USP47 influences the stability of YB-1 and found that transient transfection of USP47 prolonged the half-life of YB-1 in K562 cells (Fig. 5h). In contrast, *USP47* knockdown or P22077 treatment reduces the half-life of YB-1 (Fig. 5i and Supplementary Fig. 5b). The half-life of YB-1 is also reduced in *Usp47* knockout MEF cells (Fig. 5j). The *USP47* knockdown-induced downregulation of the YB-1 protein can be rescued by the pre-treatment of MG132, a proteasome inhibitor (Fig. 5k). Meanwhile, knockdown of USP47 has no effect on YB-1 or POLB at mRNA level and YB-1 mRNA expression is similar in normal and CML cells (Supplementary Fig. 5c, d), indicating the increased YB-1 protein levels in primary CML cells mainly attributes to the post-transcriptional regulation of YB-1. Moreover, YB-1 expression is restored in *Usp47*^−*/*−^ MEFs after the reintroduction of Usp47 (Fig. 5l). These findings suggest that YB-1 serves as a novel substrate for USP47.

### YB-1 contributes to USP47-mediated DNA damage repair in CML cells

**Fig. 6:**
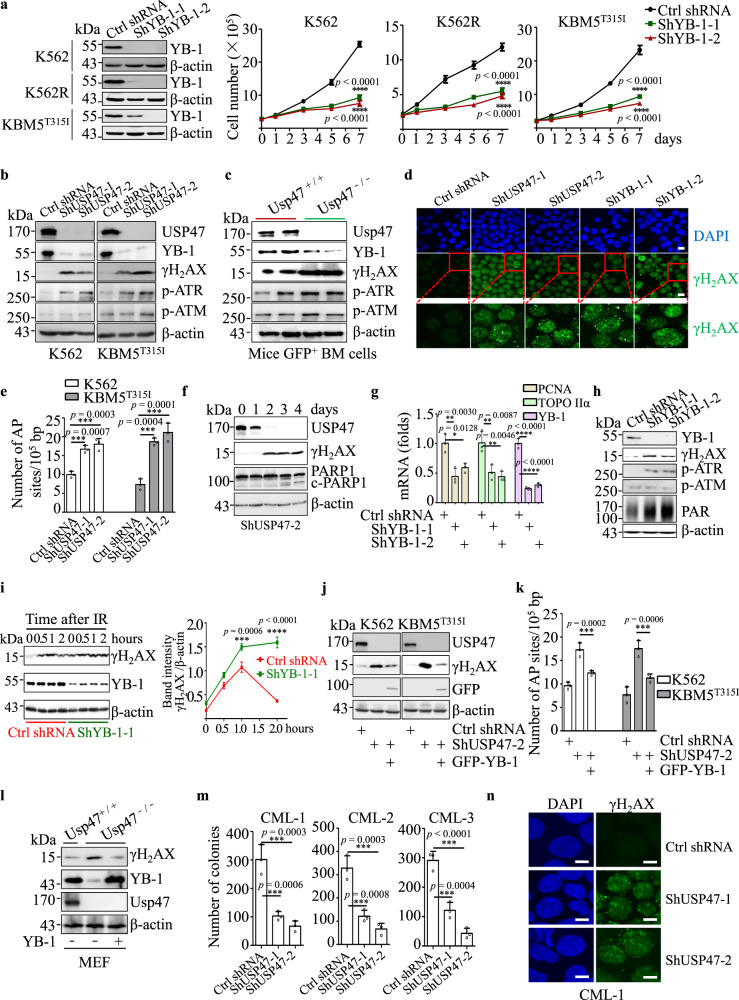
YB-1 contributes to USP47-mediated DNA damage repair in CML cells. **a** *YB-1* was silenced in K562, K562R, and KBM5^T315I^ cells with a retroviral transduction system, and viable cells were counted in the transfected cell lines at different times (*n* = 3 biologically independent samples per group). Data are mean ± s.d. *p*-values were analyzed by two-way analysis of variance (ANOVA). ****p < 0.0001. **b** DNA damage-related protein expression was measured by western blot after *USP47* knockdown in the K562 and KBM5^T315I^ cells. **c** GFP^+^ BM mononuclear cells were collected from *Usp47*^*+/+*^ and *Usp47*^*−/−*^ CML mice, and the indicated proteins were examined by western blot. **d** Immunofluorescence staining of γH_2_AX foci in the *USP47* or *YB-1* knockdown K562 cells. Scale bars, 20 μm. **e** The number of AP sites (DNA damage quantification) was detected in genomic DNA after *USP47* knockdown in the K562 and KBM5^T315I^ cells (*n* = 3 biologically independent samples per group). Data are mean ± s.d. *p*-values were analyzed by one-way analysis of variance (ANOVA). ***p < 0.001. **f** Time-course analysis of the *USP47* knockdown-induced DNA damage response in K562 cells and cleaved-PARP1 by western blot. **g** *YB-1*, *PCNA*, and *TOPO IIα* mRNA levels after *YB-1* knockdown at day 7 in K562 cells (*n* = 3 biologically independent samples per group). Data are mean ± s.d. *p-*values were analyzed by one-way analysis of variance (ANOVA). *p < 0.05; **p < 0.01; ****p < 0.0001. **h** DNA damage-related protein expression was measured by western blot after *YB-1* knockdown in the K562 cells. **i** The time-course of DNA damage is shown by γH_2_AX expression after irradiation (IR, 3 Gy) in the control and YB-1 stably knockdown KBM5^T315I^ cells. Band intensity (γH_2_AX relative to β-actin) is shown by the histogram (*n* = 3 biologically independent samples per group). Data are mean ± s.d. *p-*values were analyzed by two-way analysis of variance (ANOVA). ***p < 0.001; ****p < 0.0001. **j** Exogenous YB-1 protein was ex*p*ressed in the cell line with stably knocked down *USP47*, the expression of γH_2_AX and exogenous YB-1 (GFP tag) was detected by western blot. **k** The number of AP sites was measured as described above (*n* = 3 biologically independent samples per group). Data are mean ± s.d. *p-*values were analyzed by one-way analysis of variance (ANOVA). ***p < 0.001. **l** γH_2_AX expression in *Usp47*^*−/−*^ MEFs was measured by western blot after the reintroduction of YB-1. **m**, **n** *USP47* was knocked down in primary CML CD34^+^ cells, then the cells were cultured in a stem cell colony formation medium. The colonies (**m**) were counted on day 14 (*n* = 3 biologically independent samples per group). Data are mean ± s.d. *p-*values were analyzed by one-way analysis of variance (ANOVA). ***p < 0.001. Immunofluorescence staining of γH_2_AX foci (**n**) was performed (*n* = 3 biologically independent samples per group). Scale bars, 20 μm. Source data are provided as a Source Data file.

We examined whether YB-1, a well-known multifunctional transcription factor and an oncoprotein in cancers^39–42^, contributes to the proliferation suppression of CML cells induced by USP47 inhibition. As expected, YB-1 knockdown significantly inhibits the proliferation of K562, K562R, and KBM5^T315I^ cells (Fig. 6a). In agreement with previous studies that USP47 is involved in the DNA damage repair pathway^26,43^, *USP47* knockdown significantly increases γH_2_AX and ATR phosphorylation (Ser428) in CML cells (Fig. 6b). Furthermore, *Usp47* knockout significantly enhances the expression of γH_2_AX in GFP^+^ BM cells from BCR-ABL^T315I^-induced CML mice (Fig. 6c). These results were further confirmed by γH_2_AX foci and the number of AP sites (DNA damage quantification analysis) (Fig. 6d, e). Time-course analysis of USP47 knockdown-induced DNA damage response shows that, with the decrease of USP47, γH_2_AX increases, followed by the cleavage of PARP1, indicating that DNA damage response is not a secondary effect of cells already committed to apoptosis (Fig. 6f and Supplementary Fig. 6a). Consistent with previous findings that YB-1 regulates PCNA, epidermal growth factor (EGF), and DNA topoisomerase II α (TOPO IIα)^44–46^, YB-1 knockdown reduces the mRNA expression of PCNA and TOPO IIα (Fig. 6g and Supplementary Fig. 6b), indicating YB-1 is involved in DNA damage response in CML cells. The role of YB-1 in DNA damage repair was further validated as we found that YB-1 knockdown induces the increase of ATR phosphorylation (Ser428), PAR, and γH_2_AX in CML cells (Fig. 6h and Supplementary Fig. 6c). YB-1 knockdown also causes γH_2_AX foci in CML cells (Fig. 6d and Supplementary Fig. 6d). γH_2_AX clearance is also significantly slower in irradiation-induced *YB-1* knockdown cells than in control cells (Fig. 6i). To assess the contribution of YB-1 and Polβ to USP47-mediated DNA damage repair in CML cells, we overexpressed USP47 in YB-1 and/or *POLB* knockdown K562 and KBM5^T315I^ cells. As shown in Supplementary Fig. 6e, knockdown of *POLB* cannot increase γH_2_AX expression. USP47 overexpression in YB-1 and/or Polβ depletion cells cannot abrogate γH_2_AX expression, indicating that YB-1 contributes more to USP47-mediated DNA damage repair than Polβ in CML cells (Supplementary Fig. 6f). As expected, overexpressing YB-1 attenuates DNA damage response induced by *USP47* knockdown, as evidenced by the decrease of γH_2_AX expression and the number of AP sites (Fig. 6j, k). Also, YB-1 overexpression in *Usp47*^−/−^ MEFs attenuates the upregulation of γH_2_AX (Fig. 6l). Additionally, the utilization of AZD6738 (50 nM), an ATR pathway inhibitor, partially inhibits *USP47* knockdown-induced suppression of cell viability in CML cells (Supplementary Fig. 6g). As DNA damage repair plays an essential role in the maintenance or generation of CML stem/progenitor cells^47,48^, we hypothesized that DNA damage induced by *USP47* knockdown might be vital to CML stem/progenitor cells. In agreement with our hypothesis, *USP47* knockdown significantly decreases the colony formation ability of primary CML CD34^+^ cells (Fig. 6m). The number of γH_2_AX foci also increases after *USP47* knockdown in primary CD34^+^ cells (Fig. 6n). The results indicate that YB-1 is involved in USP47-mediated DNA damage repair in CML cells.

### P22077 exerts remarkable toxicity on CML cells and CML LSCs in vitro and in vivo

**Fig. 7:**
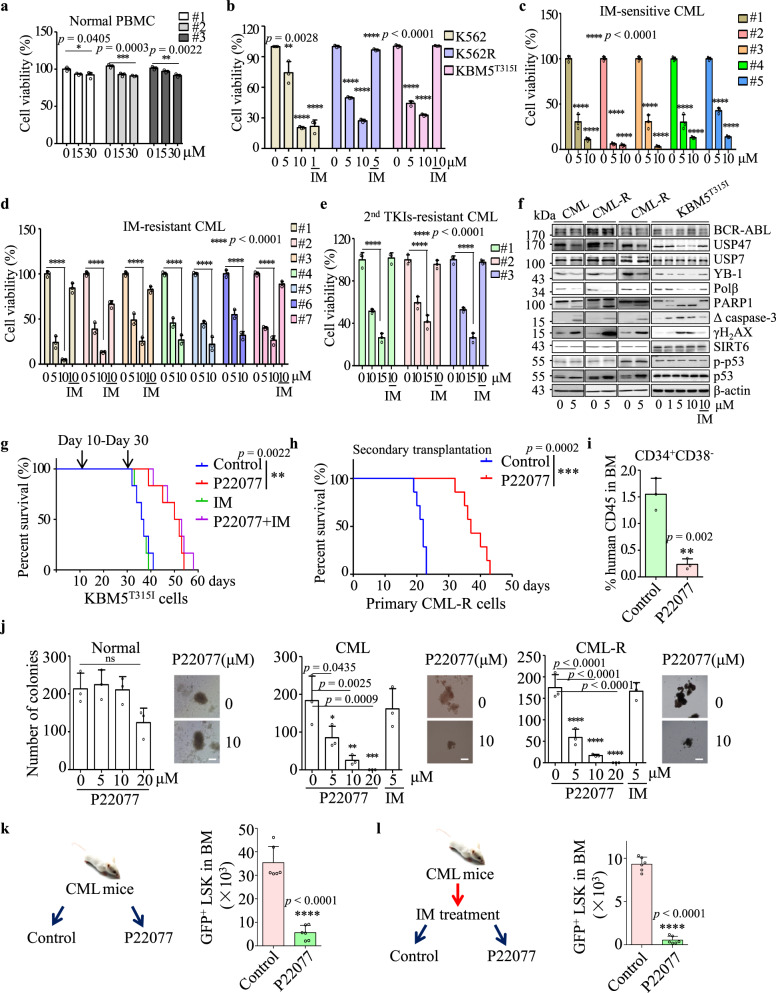
P22077 shows remarkable toxicity on CML cells and CML leukemia stem/progenitor cells in vitro and in vivo. **a**–**e** Peripheral blood mononuclear cells (PBMC) from normal donors (**a**) (*n* = 3 samples examined over three independent experiments), CML cell lines (**b**) (*n* = 3 cells examined over three independent experiments), and BM mononuclear cells from IM-sensitive (**c**) (*n* = 5 samples examined over three independent experiments) and IM-resistant (**d**) (*n* = 7 samples examined over three independent experiments) CML patients were treated with different concentrations of P22077 or IM for 48 h. Cell viability was measured by CCK8 assay. **e** BM mononuclear cells resistance to the second-generation TKIs (dasatinib, nilotinib, bosutinib) from CML patients were treated with P22077 and IM for 48 h (*n* = 3 samples examined over three independent experiments). Cell viability was measured by CCK8 assay. Data are presented as mean ± s.d. *p-*values were analyzed by one-way analysis of variance (ANOVA), *p < 0.05; **p < 0.01; ***p < 0.001, ****p < 0.0001. **f** Primary CML (IM-sensitive), CML-R (IM-resistant) BM mononuclear cells and KBM5^T315I^ cells were treated with P22077 for 48 h. Cell lysates from the indicated cells were extracted and subjected to immunoblotting with indicated antibodies. **g** P22077 (30 mg/kg/day), IM (50 mg/kg/day), P22077 (30 mg/kg/day) in combination with IM (50 mg/kg/day) or vehicle were given to mice transplanted with KBM5^T315I^ cells by intraperitoneal injection (*n* = 6 biologically independent samples per group) from day 15 to day 30. **p < 0.01, by Mantel-Cox-log-rank test. **h**, **i** Human CD45 cells from CML BM (patient-derived xenograft model) were transplanted into two groups of B-NDG mice. The survival time of the mice (**h**) (*n* = 7 biologically independent samples per group) was determined, and the percentages of CD34^+^CD38^−^ cells in human CD45 cells (**i**) (*n* = 3 biologically independent samples per group) from BM were measured. Data are mean ± s.d. *p-*values were analyzed by Mantel-Cox-log-rank test (**h**) and two-sided Student’s *t*-test (**i**). **p < 0.01; ***p < 0.001. **j** CD34^+^ cells derived from normal (*n* = 3 samples examined over three independent experiments), primary IM-sensitive CML (*n* = 3 samples examined over three independent experiments), and primary IM-resistant CML BM mononuclear cells (*n* = 3 samples examined over three independent experiments) were treated with different concentrations of P22077. The colonies were counted on day 14. Scale bars, 100 μm. Data are mean ± s.d. *p-*values were analyzed by one-way analysis of variance (ANOVA). *p < 0.05; **p < 0.01; ***p < 0.001; ****p < 0.0001; ns no significant. **k** Wild-type mice received BCR-ABL retroviral transplantation for 21 days, and the mice were treated with control or P22077 by intraperitoneal injection for 14 days. GFP^+^LSK cells from the BM of the two groups (*n* = 6 biologically independent samples per group) were evaluated by FACS. Data are mean ± s.d. *p-*values were analyzed by two-sided Student’s *t*-test. ****p < 0.0001. **l** Wild-type mice received BCR-ABL retroviral transplantation for 21 days, and the mice were trea*t*ed with IM for 40 days. Mice were divided into two groups (*n* = 6 biologically independent samples per group). One group received P22077, and the other group received the vehicle and used as the control. After 12 days, the number of GFP^+^LSK cells in the BM of the mice was examined. Data are mean ± s.d. *p-*values were analyzed by two-sided Student’s *t*-test. ****p < 0·0001. Source data are provided as a Source Data file.

P22077, a dual inhibitor of USP7/USP47, was used to assess the function of USP47 in CML cell lines and primary CML cells. As depicted in Fig. 7a, P22077 exhibits low toxicity to mononuclear cells from normal PB, while in contrast, P22077 inhibits the proliferation of both IM-sensitive and IM-resistant CML cell lines and primary CML cells in a dose-dependent manner (Fig. 7b–d). Notably, P22077 shows evident cytotoxicity to BM mononuclear cells derived from CML patients resistant to second-generation TKIs (Fig. 7e). In both primary IM-sensitive and IM-resistant CML cells, P22077 treatment decreases YB-1 but increases the expression of γH_2_AX and the cleavage of caspase-3 and PARP1, indicating the activation of apoptosis (Fig. 7f). In addition, USP47 is positively correlated with the expression of YB-1 in primary CML cells, and CML cells with higher levels of USP47 are more sensitive to P22077 treatment than the cells with lower levels of USP47 (Supplementary Fig. 7a). We then evaluated the effect of P22077 on KBM5^T315I^ cells using a B-NDG mouse CML model. The B-NDG mice injected with KBM5^T315I^ cells by tail vein were divided into four groups and treated with the vehicle, IM, P22077, and P22077 plus imatinib, respectively. P22077 significantly prolongs the survival of CML mice, while P22077 combined with IM has no significant enhancement effect on P22077 (Fig. 7g). More importantly, using CML patient-derived xenograft model established from a TKI-resistant CML patient, we found that P22077 treatment could significantly prolong the survival of mice (Supplementary Fig. 7b) and reduce the percentages of CD34^+^CD38^−^ cells in the secondary bone marrow transplantation (Fig. 7h, I). Given the important role of USP47 in CML stem/progenitor cells, we investigated the effect of P22077 on primary CD34^+^ CML cells. P22077 exerts no cytotoxicity on normal CD34^+^ cells, but significantly inhibits the colony-forming activity of CD34^+^ cells from de novo and IM-resistant CML patients (Fig. 7j). Moreover, P22077 significantly decreases the number of CML stem/progenitor cells in a BCR-ABL-induced CML mouse model (Fig. 7k). P22077 treatment significantly eliminates the number of CML GFP^+^LSKs in the MRD mouse model (Fig. 7l). These results indicate that P22077 exerts cytotoxicity markedly against CML cells, including CML stem/progenitor cells.

## Discussion

TKI resistance and CML leukemia stem cells remain the major challenges in the treatment of BCR-ABL^+^ CML. In this study, we demonstrate that USP47 plays a critical role in the proliferation of TKI-sensitive, TKI-resistant, and CML stem/progenitor cells both in vitro and in vivo. As a substrate of USP47, YB-1 contributes to USP47-mediated DNA damage repair in CML cells. Hence, targeting USP47 is a promising strategy to overcome TKI resistance and eradicate CML stem/progenitor cells.

The role of USP47 in cancer is cancer type-dependent. It can function as an oncoprotein in gastric cancer^49^, colorectal^29,30^, ovarian cancer^50^, and a tumor suppressor in medulloblastoma^36^. Our study reveals that USP47 is essential in the pathogenesis of CML. We show that knockout of *Usp47* significantly prevents BCR-ABL or BCR-ABL^T315I^-induced leukemogenesis and reduces the number of GFP^+^LSK cells in CML mice, indicating that *Usp47* is critical in the development of BCR-ABL-induced CML. Moreover, targeting USP47 is effective in CML cells regardless of the presence of BCR-ABL with or without kinase domain mutation. CML cells derived from all analyzed CML patients (irrelevant to BCR-ABL mutation or TKI resistance) exhibit similar sensitivity to USP47 inhibition-induced cytotoxicity. Furthermore, USP47 inhibition subdues the proliferation of K562, K562R (without BCR-ABL mutation and insensitive to IM), and KBM5^T315I^ (with BCR-ABL T315I mutation and insensitive to IM) CML cell lines both in vitro and in vivo. Therefore, our study suggests that USP47 is a promising therapeutic target in CML treatment, especially for CML patients with TKI resistance.

Consistent with previous reports^26,43^, we found that USP47 is involved in the DNA damage repair in CML cells. We further demonstrated that YB-1, a novel substrate of USP47, contributes to USP47-mediated DNA damage repair. In agreement with our findings, it has been reported that YB-1 is associated with cancer progression and drug resistance in multiple cancers by stimulating cell proliferation and promoting replicative immortality, genomic instability, and metastasis^39–42,51–53^. Several studies reported that YB-1 is also involved in base excision and mismatch repair pathways by interacting with multiple DNA repair proteins, such as DNA ligase IIIα^54^, APE1^55^, MSH2^56^, and PCNA^57^. In CML, BCR-ABL compromises the fidelity of nucleotide excision repair and homologous recombination repair, thus promoting survival and resulting in the genomic instability of CML cells, especially LSCs^11,58–60^. Therefore, it is reasonable that in CML cells, BCR-ABL induces DNA damage, while USP47 and YB-1 are required to repair DNA damage to ensure cell survival.

Another important finding is that P22077 is effective in TKI-sensitive and TKI-resistant CML cells, regardless of the presence of T315I mutation. Interestingly, in addition to inhibiting the activity of USP47, P22077 considerably reduces the protein level of USP47 in CML cells. P22077 may promote the degradation of USP47 by inhibiting the auto-deubiquitinating process of USP47^28^, which is supported by the finding that MG132 reverses P22077-induced USP47 degradation (Supplementary Fig. 7c). It has been reported that P22077 also inhibits the activity of USP7 and USP10. To identify the specific USPs targeted by P22077 in CML cells, we measured the expression of substrates of USP7 and USP10, including p53, a substrate of both USP7 and USP10^61,62^, and SIRT6, a substrate of USP10^63^. We found that P22077 increases p53 expression (but not phosphorylated p53, ser15) in primary CML cells but does not affect SIRT6 expression. However, both p53 and SIRT6 expressions are not increased by P22077 treatment in KBM5^T315I^ cells (Fig. 7f). Also, USP10 or USP7 knockdown does not affect the expression of YB-1 (Supplementary Fig. 7d). Moreover, P22077 still strongly inhibits the viability of *USP7* or *USP10* stably knockdown K562 cells (Supplementary Fig. 7e). Therefore, we deem that USP47 is the main effector for P22077 to combat CML. In addition, it should be noted that, mice treated with P22077 (30 mg/kg) for 2 weeks did not show health problems such as weight loss (Supplementary Fig. 7f), which was consistent with the previous reports^64^. These data indicate P22077 is a promising candidate compound for the treatment of TKI-sensitive and TKI-resistant CML. Nevertheless, further investigations are still needed to develop USP47-specific inhibitors for CML treatment.

Based on the fact that TKI reduces the expression of USP47 in CML cells and overexpression of BCR-ABL upregulates the expression of USP47 in 32D cells, we demonstrate that BCR-ABL upregulates the expression of USP47. However, BCR-ABL may not be the only regulator of USP47. First, the activation of RAS/ERK, which is independent of BCR-ABL, can regulate the expression of USP47^65,66^. Second, USP47 is highly expressed in several types of BCR-ABL-negative cell lines, including colorectal cancer cells and gastric cancer cells^30,49^. Therefore, USP47 may be regulated through both BCR-ABL-dependent and -independent mechanisms.

In summary, we demonstrate that USP47 is a target for CML treatment and targeting USP47 is a promising strategy for overcoming TKI resistance and eradicating leukemia stem/progenitor cells in CML.

## Methods

### Reagents and antibodies

The p-Bcr (WB, 1:1000, #3901, Lot 1), c-Abl (1:1000, #2862, Lot 13), p-CrkL (1:1000, #3181, Lot 7), CrkL (1:1000, #3182, Lot 5), p-ERK (1:1000, #9101, Lot 26), ERK (1:1000, #9102, Lot 25), p-P38 MAPK (1:1000, #9215, Lot 19), p-JNK MAPK (1:1000, #9251, Lot 10), p-AKT (1:1000, #4060, Lot 30), caspase-3 (1:1000, #9661, Lot 45), γH_2_AX (1:1000, #9718, Lot 1), p-p53 (1:1000, #9284), p-ATR (1:1000, #2853, Lot 9), p-ATM (1:1000, #4526, Lot 14), β-catenin (1:1000, #9587, Lot 2) and PAR (1:1000, #83732) antibodies were purchased from Cell Signaling Technology (Danvers, MA, USA). The USP47 (1:500, sc-100633, Lot J1314), STAT5 (1:500, sc-74442), USP14 (1:500, sc-100630, Lot J3013), Ubiquitin (1:500, sc-8017, Lot H0409), GFP (1:500, sc-9996, Lot J0813), PARP1 (1:500, sc-56197), PCNA (1:500, sc-53407), p53 (1:500, sc-126) antibodies were purchased from Santa Cruz Biotechnology (Santa Cruz, CA, USA). Antibodies for Polβ (1:1000, #18003-1-AP, Lot 00009472), YB-1 (1:1000, #20339-1-AP, Lot 00046294), Flag tag (1:1000, #66008^−^2-Ig), USP10 (1:1000, #19374-1-AP, Lot 00015015), TOPOIIα (1:1000, #20233-1-AP), SIRT6 (1:1000, #13572-1-AP, Lot 00004562), β-actin (1:1000, #HRP-66009) were purchased from Proteintech Group, Inc. (Wuhan, CHN). The USP7 (1:1000, #A300-033A) antibody was purchased from Bethyl Laboratories Inc. (Montgomery, TX, USA). The USP16 (1:1000, GTX16439, Lot 39631) antibody was purchased from GeneTex Inc. (Irvine, CA, USA). The above antibodies were used in western blot. Lineage Antibody Cocktail (1:200, #88-7772-72), sca-1 (1:200, #25-5981-82), c-kit (1:200, #17-1171-82), CD34 (1:200, #11-0349-42), CD38 (1:200, #12-0389-42) and CD45 (1:200, #17-0459-42) for FACS analysis were purchased from ebioscience Inc. (San Diego, CA, USA). Gr-1 antibody (1:200, #130-119-794) was purchased from Miltenyi Biotec (Auburn, CA, USA). CD45 antibody (1:1000, #GB14038) for IHC was purchased from ServiceBio (Wuhan, CHN). Flag-bead (M20018) was purchased from Abmart (Shanghai, CHN). EasySep Release Human CD45 Positive Selection Kit for Humanized Mice was purchased from STEMCELL (100-0107, Vancouver, BC, Canada). Imatinib (S2475), P22077 (S7133), and MG132 (S2619) were purchased from Selleck Chemicals (Houston, TX, USA). AZD6738 (T3338) was purchased from Target Molecule Corp. (Shanghai, CHN). Pan-RAS-IN-1 (HY-101295) was purchased from MedChemExpress LLC. (Monmouth Junction, NJ, USA). The Cell Cycle Detection Kit (340242) was purchased from BD Biosciences (San Jose, CA, USA). The Caspase-3 Activity Assay Kit (C1115) was purchased from Beyotime Biotechnology (Jiangsu, CHN).

### Cell culture

The murine IL-3-dependent myeloid cell line 32D obtained from ATCC (CRL-11346) was maintained in RPMI-1640 (21875109; Gibco. Grand Island, New York, USA) supplemented with 10% fetal bovine serum (FBS) (900-108; Gemini Bio-Products Inc. Sacramento, USA) and 10% WEHI-3B conditional medium containing IL-3; their counterparts transformed with p210^BCR−ABL^ were maintained in RPMI-1640 (11875; Gibco) supplemented with 10% FBS. The IM-sensitive K562 cells obtained from ATCC (CRL-243) and IM-resistant cell lines K562R cells obtained from ATCC (CRL-3344), respectively, were cultured in RPMI-1640 (11875; Gibco) supplemented with 10% FBS. K562R cells were cultured in 1 μM IM to maintain their drug-resistant status. KBM5^T315I^ cells^67^ were kindly provided by Jingxuan Pan in Sun Yat-sen University and were cultured in IMDM (12440061; Gibco) supplemented with 10% heat-inactivated FBS, penicillin (50 U/ml)/ streptomycin (50 μg/ml) (E607011; Sangon Biotech Co, Shanghai, CHN). All cells were cultured at 37 °C in a humidified atmosphere of 5% CO_2_. HEK293T cells were obtained from ATCC (CRL-3216) and cultured in DMEM (11971025, Gibco) with 10% FBS. All cell stocks were routinely tested for mycoplasma contamination and showed negative.

### Primary cells

BM samples were obtained from healthy donors and patients with CML (from 2011 to 2019) at different clinical stages or at drug resistance admitted to Shanghai Jiao Tong University School of Medicine affiliated Ruijin Hospital, Xinhua Hospital, Shanghai First People’s Hospital, Shanghai 9th People’s Hospital and Shanghai Tongren Hospital. The details of patients were provided in Supplementary Table 1. Mononuclear cells were isolated by Ficoll-Paque Plus density gradient media (GE Life Sciences, Chicago, IL, USA). Informed consent was obtained from all patients in accordance with the Declaration of Helsinki. All manipulations were approved by the Medical Ethic Committee of Shanghai Jiao Tong University School of Medicine.

### RNA interference

ShRNA oligonucleotides targeting *USP47*, *BCR-ABL*, *YB-1*, *STAT5*, *USP7*, and *USP10* were designed and synthesized (Supplementary Table 2). For gene silencing, *USP47*, *BCR-ABL*, *YB-1*, *STAT5*, *POLB*, *USP7*, and *USP10*-specific ShRNA and control ShRNA were cloned into the pSIREN-RetroQ vector. The retrovirus supernatant was packaged in HEK293T cells by cotransfecting with pSIREN-RetroQ, VSVG and gag-pol. The viral supernatant was collected after transfection for 48 h. The cells were treated with viral supernatant and polybrene (4 μg/ml) for 48 h.

### Lentiviral transduction in CML cells

Lentiviruses were produced by transient transfection in HEK293T cells using pLVX-ZsGreen-IRES-YB-1 or pLVX-ZsGreen-IRES-USP47 plasmid, envelope plasmid pMD2.G, and packaging plasmid psPAX2. CML cells were infected twice by spinoculation (1500 × *g*, 60 min, room temperature) with virus-containing supernatants.

### Cell cycle analysis

USP47-specific knockdown and control cells were collected separately. Then, the cells were rinsed with cold PBS and fixed in 75% ethanol at −20 °C for at least 24 h. Subsequently, the cells were rinsed again with cold PBS for two times, incubated with RNAase (10 μg/mL) at 37 °C for 30 min and stained with propidium iodide (PI; 50 μg/mL). Flow cytometry (BD FACS Calibur) was used to examine the percentages of cells at different cell cycle stages.

### Immunoprecipitation and immunoblot analysis

Whole-cell extracts were prepared with lysis buffer (P0013; Beyotime, Jiangsu, CHN), then they were incubated with the appropriate antibody overnight at 4 °C. Protein A&G beads (sc-2003; Santa Cruz Biotechnology, Santa Cruz, CA, USA) were added, and the incubation was continued for 4 h at 4 °C. Beads were washed three times with PBS buffer. The bound proteins were then separated by sodium dodecyl sulphate–polyacrylamide gel electrophoresis (SDS-PAGE), transferred to a nitrocellulose membrane, and probed with the appropriate antibodies. The image was visualized using a chemiluminescence imaging analyzer (GE, ImageQuant LAS 4000).

### Mass spectrometry analysis

Immunoprecipitation samples were subjected to SDS-PAGE and visualized with colloidal Coomassie blue. Two obvious bands were cut out and digested by sequencing-grade modified trypsin (Promega). The tryptic peptides were analyzed by liquid chromatography with tandem mass spectrometry ((LC-MS/MS)) (liquid chromatography coupled with mass spectrometry). The MS experiments were performed on an LTQ orbitrap “XL” mass spectrometer assembled by an Easy-nLC 1000 via an Easy Spray (Thermo Fisher Scientific). The peptide mixture was separated in a 50-cm-column (inner diameter = 0.075 mm) packed with C18 2-mm Reversed Phase resins (PepMap RSLC) using 300-min linear gradient elution, from 95% solvent A (0.1% formic acid and 2% acetonitrile within 98% water) to 35% solvent B (0.1% formic acid and 2% water within 98% acetonitrile) at a flow rate of 200 nl/min. All MS/MS spectra were searched against the uniprot human database by Mascot Searching engine (Matrix Science, London, UK). Finally, the validated peptides and protein identification were exported by Scaffold (Proteome Software Inc., Portland, OR) using the Scaffold Local FDR algorithm.

### Quantitative real-time PCR (RT-PCR)

Total RNA was extracted using Trizol reagent (Invitrogen Corp. Waltham, MA, USA), and cDNA was prepared using a Reverse Transcriptase kit (Takara Bio Inc. Shiga, Japan). SYBR Master Mix (Roche Diagnostics Corporation, Indianapolis, IN, USA) and the Applied Biosystems Step One PlusTM detection system (ABI 7900) were used for real-time quantitative PCR. The DUBs primer sequences (Supplementary Table 3) and specific primer sequences (Supplementary Table 4) were used to analyze gene expression.

### CML mouse model

*Usp47* knockout (*Usp47*^−*/*−^) mice (C57BL/6 J background) were obtained from MMRRC (Mutant Mouse Resource & Research Centers, USA). To generate the *Usp47*^−*/*−^ mouse with BALB/c background, we backcrossed these mice to the BALB/c mice for over 10 generations. All subsequent *Usp47*-deficient (−/−) or wild-type (WT, + / + ) mice used in this study were generated from mating with littermates. The *Usp47* genotype was determined by PCR analysis. The primers used were the following: β-Geo (580 bp) 5′-CAA ATG GCG ATT ACC GTT GA−3′, 5′-TGC CCA GTC ATA GCC GAA TA-3′; Usp47 (907 bp) 5′-CTT CAC CTG TTC AAA TCC TCC G-3′, 5′-GTT CCT TTC TGT TCA TAC CCG ATG-3′. The retroviral plasmid MIGR1 carrying the p210^BCR−ABL^ or T315I mutation p210^BCR−ABL^ was prepared by transient transfection with the Ecopack construct in HEK293T cells. BM cells from 5-fluorouracil-treated (200 mg/kg) *Usp47*^*+/+*^ or *Usp47*^−*/*−^ BALB/c donor mice (8-week) were transduced twice with *BCR-ABL* retrovirus in the presence of interleukin-3 (IL-3), interleukin-6 (IL6) and stem cell factor (SCF). Wild-type recipient mice were subjected to 7 Gy γ-irradiation and followed by injection with infected BM cells (1 × 10^6^ per mouse) via tail vein injection. After transplantation, the incidence of CML in the recipient mice was observed and recorded.

The mice were bred in an equipped animal facility with the temperature at 20–25 °C and humidity at 30–70%, with a 12 h light–dark cycle and ad libitum access to regular chow diet and water. All animal procedures were approved by the committee for the humane treatment of animals at Shanghai Jiao Tong University School of Medicine. The study was compliant with all of the relevant ethical regulations regarding animal research.

### B-NDG mouse model

Six-week-old NOD-SCID IL-2 receptor gamma (B-NDG) null female mice were obtained from Jiangsu Biocytogen Co., Ltd. (Nantong, CHN). KBM5^T315I^ cells (4 × 10^6^) transduced with control ShRNA or USP47 ShRNA were injected into the tail vein of mice (*n* = 8, each). The survival of mice from the tail vein model was analyzed and is shown with a Kaplan-Meier survival plot. The spleen from the sacrificed mice was measured to determine whether the mice had splenic enlargement. For drug administration experiments, KBM5^T315I^ cells (2 × 10^6^) were transplanted via tail vein injection into 6-week-old B-NDG mice. Between 15 and 30 days, the mice were treated through tail vein injection of vehicle (10% DMSO + 10% BASF-ELP + 10% 1,2-Propanediol + 70% physiological saline) (*n* = 6), P22077 (dissolved in the vehicle) (intraperitoneally, 30 mg/kg, *n* = 6), IM (intraperitoneally, 50 mg/kg, *n* = 6) or P22077 + IM (intraperitoneally, *n* = 6). The survival time of the mice was determined. Primary CML-R cells (5 × 10^6^) were transplanted via tail vein injection into 6-week-old B-NDG mice. Between 30 and 45 days, the mice were treated every day through tail vein injection of vehicle (*n* = 6), P22077 (dissolved in the vehicle) (intraperitoneally, 30 mg/kg, *n* = 6). For the secondary transplantation, human CD45 cells (2 × 10^6^) from BM were transplanted into two groups (*n* = 7) of B-NDG mice. The survival times of the mice were determined, and the percentages of CD34^+^CD38^−^ cells in human CD45 cells from BM were measured. The graphical account for FACS (BD FACS Calibur) sequential gating/sorting strategies was provided in Supplementary Fig. 8.

### Immunohistochemical and H&E staining

The samples were fixed with 4% paraformaldehyde for 2 days, then dehydrated through a graded series of ethanol and embedded in paraffin. Sections were cut and stained with haematoxylin-eosin (H&E). For immunohistochemistry, the samples were incubated overnight at 4 °C with primary antibodies after antigen retrieval in citrate buffer. The samples were incubated for 30 min with biotinylated second antibody IgG and then for 20 min with Streptavidin-HRP peroxidase. The reaction products were visualized with diaminobenzidine (DAB)-H_2_O_2_ as a substrate for peroxidase. All sections were counterstained with hematoxylin. The image was visualized using a microscope (Olympus, CKX31).

### Immunofluorescence staining

The cultured cells were fixed to a slide by a cytospin and fixed with 0.3% Triton X-100. After permeabilization with 100% cold methanol and blocking with 2% (w/v) bovine serum albumin, the cells were incubated γH_2_AX antibody (1:100 dilution) overnight at 4 °C. Then, the slides were stained with the secondary antibody and DAPI. The fluorescence signal of the cells was detected by confocal microscopy (Nikon, A1R-si).

### DNA damage quantification

Genomic DNA was isolated and purified using the QIAamp DNA Mini Kit (51304; QIAGEN, Hilden, Germany). The number of apurinic/apyrimidinic sites (AP sites) was determined using the DNA Damage Quantification Kit (DK02, DOJINDO Laboratories, Kumamoto, Japan) according to the manufacturer’s instruction.

### Cell viability assay

CML cell lines and primary CML cells were incubated with different concentrations of P22077 and/or IM for 48 h. Cell viability was assayed using a Cell Counting Kit-8 (CK04, DOJINDO Laboratories, Kumamoto, Japan) according to the manufacturer’s instructions.

### Colony-forming assay

CD34^+^ cells from BM mononuclear cells of CML or normal healthy volunteers were obtained using human CD34^+^ selection cocktail (14756, STEMCELL, Vancouver, BC, Canada). CD34^+^ cells were infected with *USP47* knockdown lentivirus for 24 h, and then the cells were seeded at 1000 cells per well of a 12-well plate. Different concentrations of P22077 and 1000 CD34^+^ cells were mixed with the stem cell medium (H4434, STEMCELL), and then continuously incubated at 37 °C in 5% humidified CO_2_. After incubation for 14 days, the colonies were counted.

### MRD mouse model

BCR-ABL retrovirus-infected mouse BM cells were transplanted into 6-week-old female wild-type mice, and the GFP^+^Gr-1^+^ cells were detected by FACS on day 21. The CML mice were treated with IM (50 mg/kg, intraperitoneally) for 40 days, and then the mice were randomly divided into two groups. One group was treated with P22077 (30 mg/kg, intraperitoneally), the other group was given vehicle as control. The effect of P22077 on GFP^+^LSKs in BM was examined by FACS after 12 days.

### Mouse xenograft model

K562 control and USP47 stably knockdown cells (8 × 10^6^) were implanted subcutaneously into the right flanks of 6-week-old female Balb/c (nu/nu) mice (Slac Laboratory Animal Co., Ltd., China). Tumor sizes were measured using calipers, and tumor volumes were calculated using a standard formula (width^2^ × length/2). Tumor cell proliferation and apoptosis were detected by hematoxylin and eosin (H&E) staining, PCNA immunohistochemical (IHC) staining, and TUNEL assays were performed.

### Homing analysis

We transduced BM from *Usp47*^−/−^ and *Usp47*^+/+^ mice (*n* = 3, each group, 8-week-old) with *BCR-ABL* retrovirus for 48 h. GFP^+^LSK cells in infected BM cells were measured by FACS. The infected BM cells (before transplantation) were then injected into lethally irradiated 7.5 Gy *Usp47*^+/+^ mice. The BM cells were collected 18 h after injection (after transplantation). The GFP^+^LSK cells were monitored by FACS. The homing efficacy was calculated by the ratio of the percentage of GFP^+^LSK cells: [GFP^+^LSK] after transplantation 18 h/ [GFP^+^LSK] before transplantation.

### Statistics and reproducibility

All statistical tests were performed using GraphPad Prism 6 (GraphPad Software Inc.). All data are presented as the mean ± sd. Student’s *t*-test was used to determine significant differences between two groups. One-way or two-way analysis of variance (ANOVA) was used to analyze the significant differences among multiple groups. For all statistical tests, *p-*values < 0.05 were considered to be statistically significant. The number of independent experiments and replicates (*n*) is indicated in the figure legends. At least three biologically independent replicates were performed for each experiment and similar results were obtained.

### Reporting summary

Further information on research design is available in the Nature Research Reporting Summary linked to this article.

## Supplementary information

Supplementary Information

Peer Review File

Reporting Summary

## Data Availability

The data supporting the findings of this study are available within the article and its supplementary materials, which are provided as a Source Data file. In addition, some raw data was generated in the Core Facility of Basic Medical Sciences of our school. Derived data supporting the findings of this study are available from the corresponding author upon reasonable request. Source data are provided with this paper.

## Source data

Source Data
