# Supplementary material for: Targeting USP47 overcomes tyrosine kinase inhibitor resistance and eradicates leukemia stem/progenitor cells in chronic myelogenous leukemia
Source: Nat Commun. 2021 Jan 4;12:51. doi: 10.1038/s41467-020-20259-0 (PMC7782553; doi:10.1038/s41467-020-20259-0)
Supplement: Supplementary file 1 — Supplementary Information [file 41467_2020_20259_MOESM1_ESM.pdf]

Targeting USP47 overcomes tyrosine kinase inhibitor resistance and eradicates leukemia stem/progenitor cells in chronic myelogenous leukemia

Supplementary Information

Contents

Supplementary Figure 1

Supplementary Figure 2

Supplementary Figure 3

Supplementary Figure 4

Supplementary Figure 5

Supplementary Figure 6

Supplementary Figure 7

Supplementary Figure 8

Supplementary Table 1

Supplementary Table 2

Supplementary Table 3

Supplementary Table 4

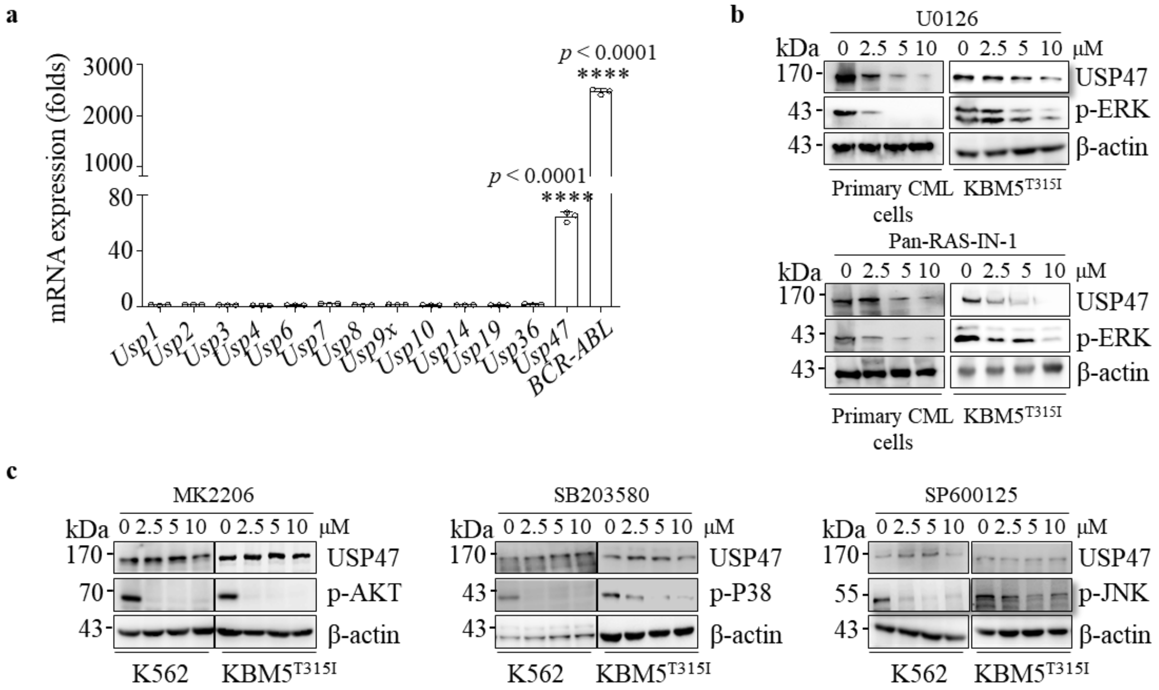

Supplementary Fig. 1 USP47 is regulated by the RAS/ERK pathway. a The mRNA expression of different USPs in vector ($32D^{MIGIR}$) and $P210^{BCR-ABL}$ stably transfected 32D ($32D^{BCR-ABL}$) cells by real-time quantitative PCR (n=3 biologically independent samples per group). Data are mean ± s.d., two-sided Student's *t*-test. ****, $p<0.0001$. **b-c** Primary CML cells, K562, and $KBM5^{T315I}$ cells were treated with indicated inhibitors for 24 hours, and the indicated proteins were detected by Western blot. Source data are provided as a Source Data file.

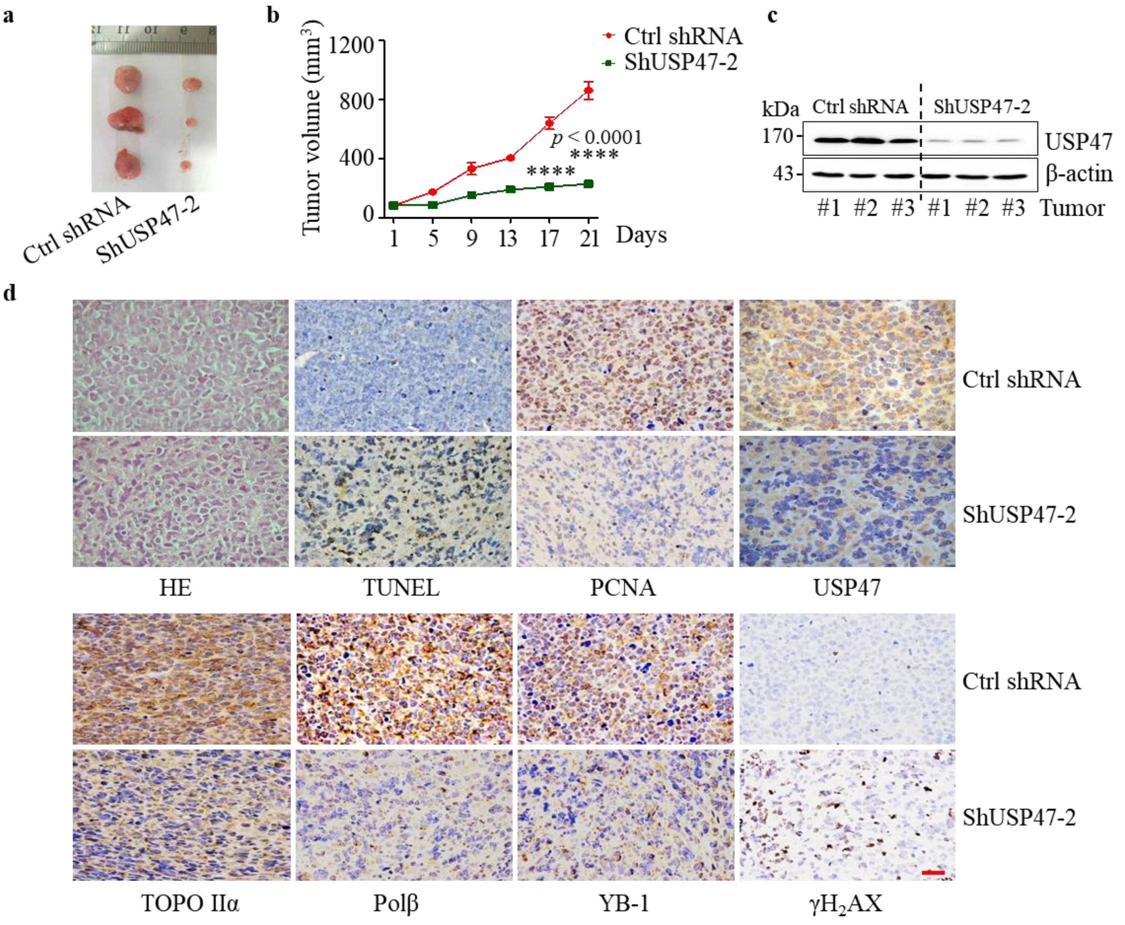

Supplementary Fig. 2 USP47 promotes CML cell survival *in vivo*. a-c USP47 knockdown K562 cells (ShUSP47-2) and control K562 cells (Ctrl ShRNA) (8×10^6) were subcutaneously transplanted into nude mice. Tumor sizes at day 21 are shown (**a**), and tumor volumes were measured every 4 days (**b**). USP47 expression was measured by Western blot in tumor cells (**c**). Data are mean ± s.d., *P* values were analyzed by two-way analysis of variance (ANOVA), ****, $p<0.0001$. **d** The expression of indicated proteins was examined by immunohistochemistry in tumor tissues. Scale bar: 50 μm. Source data are provided as a Source Data file.

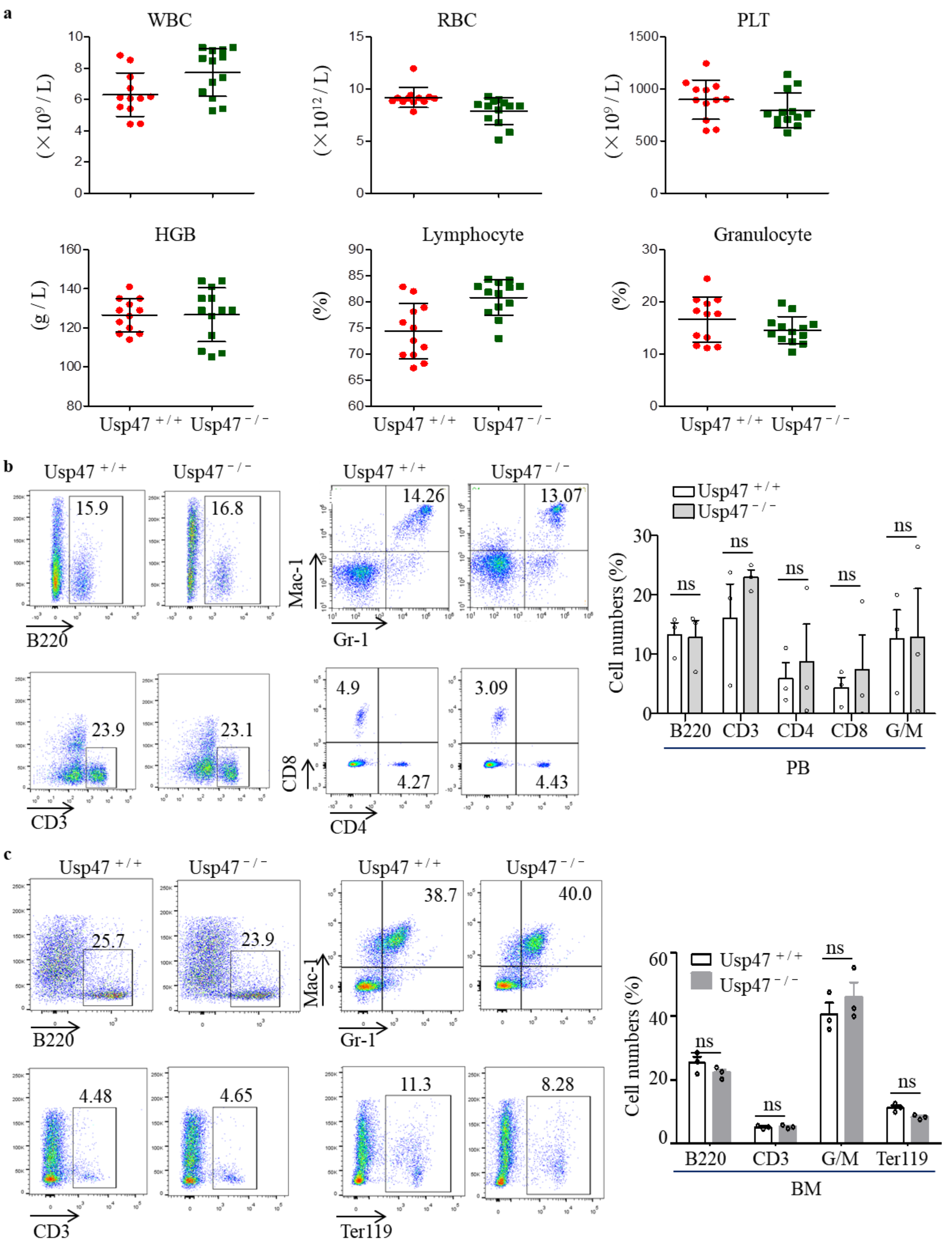

Supplementary Fig. 3 ***Usp47*** **knockout mice have normal hematopoietic system. a** Number of total white blood cells, red blood cells, granulocytes, lymphocytes, and platelets were analyzed with a blood analyzer in the PB of the *Usp47*$^{+/+}$ (n=12) and *Usp47*$^{-/-}$ (n=13)

mice. WBC, total number of white blood cells; RBC, total number of red blood cells; PLT, platelets; HGB, hemoglobin. **b, c** Percentages and number of different blood cells in PB (**b**) and BM (**c**) were evaluated. (n=3 biologically independent samples per group) B220, B cells; CD3, T cells; CD4, helper T cells; CD8, cytotoxic T cells; G/M (Gr-1/Mac-1), myeloid cells; Ter119, erythroid cells. Data are mean ± s.d., two-sided Student's *t*-test. ns, not significant. Source data are provided as a Source Data file.

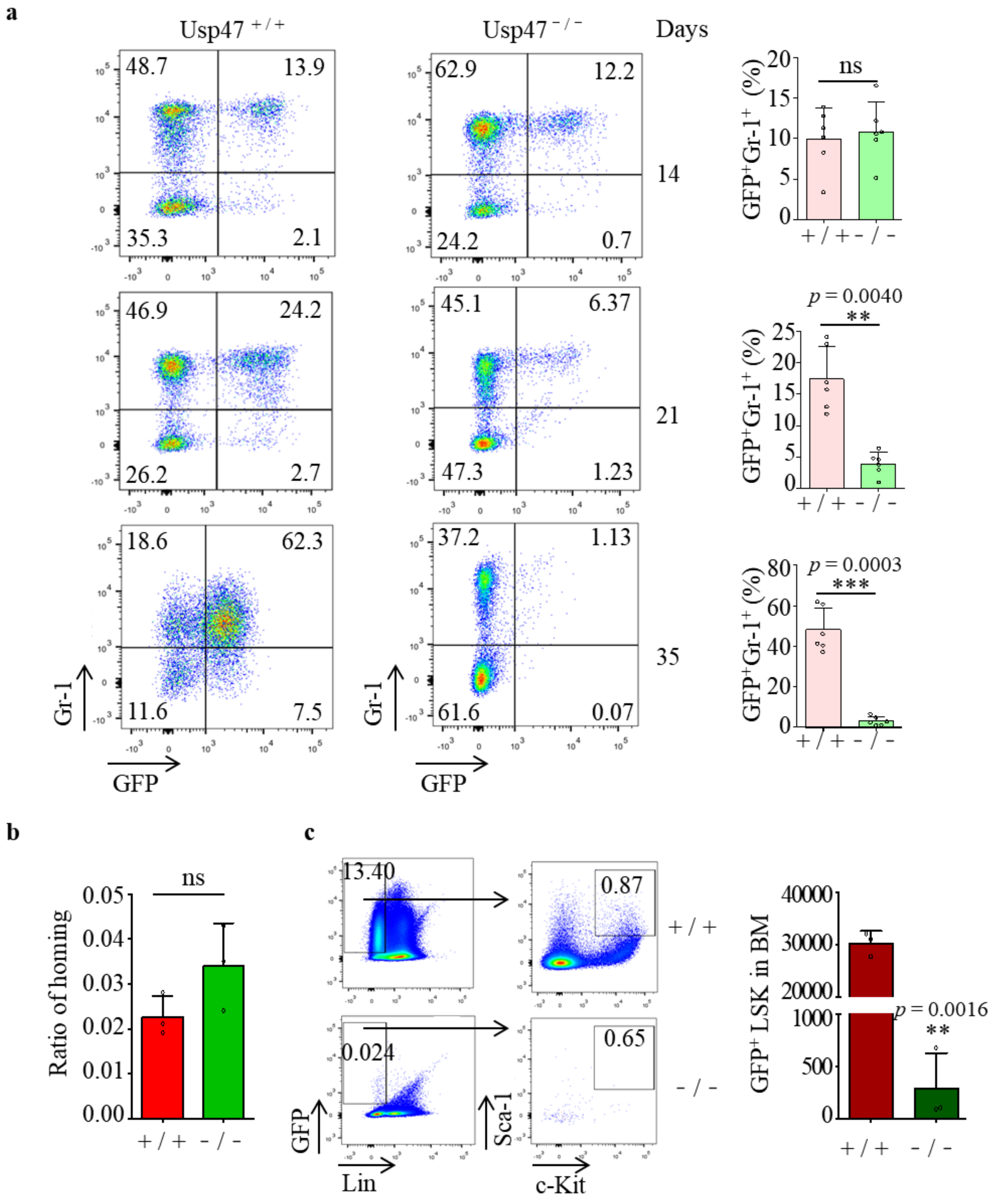

Supplementary Fig. 4 *Usp47* knockout does not affect the homing efficacy of CML LSKs but eliminates GFP$^+$LSKs in CML mice. a Number of GFP$^+$Gr-1$^+$ cells was measured in PB at different times after BCR-ABL^{T315I} retrovirus transplantation (n=6 biologically independent samples per group). Data are mean ± s.d., two-sided Student's *t*-test. **, $p<0.01$; ***, $p<0.001$;

ns, not significant. **b** *Usp47*$^{-/-}$ and *Usp47*$^{+/+}$ BM cells were infected with BCR-ABL retrovirus for 48 hours, and the percent of GFP^{+}LSK cells were measured by FACS. The infected BM cells were injected into lethally irradiated *Usp47*$^{+/+}$ mice. GFP^{+}LSK cells were monitored by FACS in BM after 18 hours. The homing efficacy was calculated by the ratio of the percentage of GFP^{+}LSK cells ([GFP^{+}LSK] after transplantation 18 h/[GFP^{+}LSK] before transplantation) in BM (n=3 biologically independent samples per group). Data are mean ± s.d., two-sided Student's *t*-test. ns, not significant. **c** At 35 days after BCR-ABL^{T315I} retrovirus transplantation, the number of GFP^{+}LSKs in BM was examined by FACS (n=3 biologically independent samples per group). Data are mean ± s.d., two-sided Student's *t*-test. **, p<0.01. Source data are provided as a Source Data file.

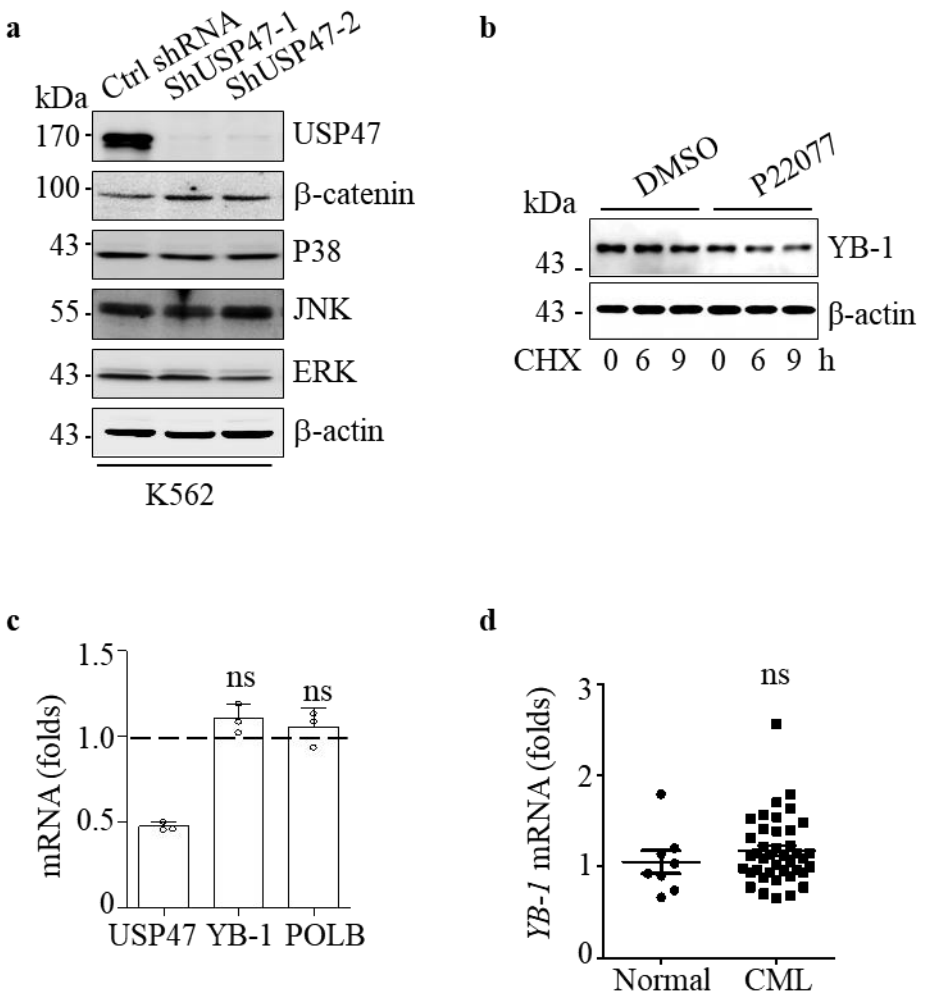

Supplementary Fig. 5 USP47 interacts with and stabilizes YB-1. a The indicated proteins were detected by Western blot in USP47 stably knockdown K562 cells. **b** K562 cells were treated with P22077 (5 µM) or DMSO for 6 hours first. The cells were then treated with cycloheximide (CHX, 10 µM) for different times; the indicated proteins were determined by Western blot. **c** The mRNA expression of *USP47*, *YB-1,* and *POLB* in USP47 stably knockdown K562 cells compared to the control cells (n=3 biologically independent samples per group). Data are mean ± s.d., two-sided Student's *t*-test. ns, no significant. **d** The expression of YB-1 in BM mononuclear cells from CML patients (n=41) compared to normal BM $CD34^+$ cells (n=8). Data are mean ± s.d., two-sided Student's *t*-test. ns, no significant. Source data are provided as a Source Data file.

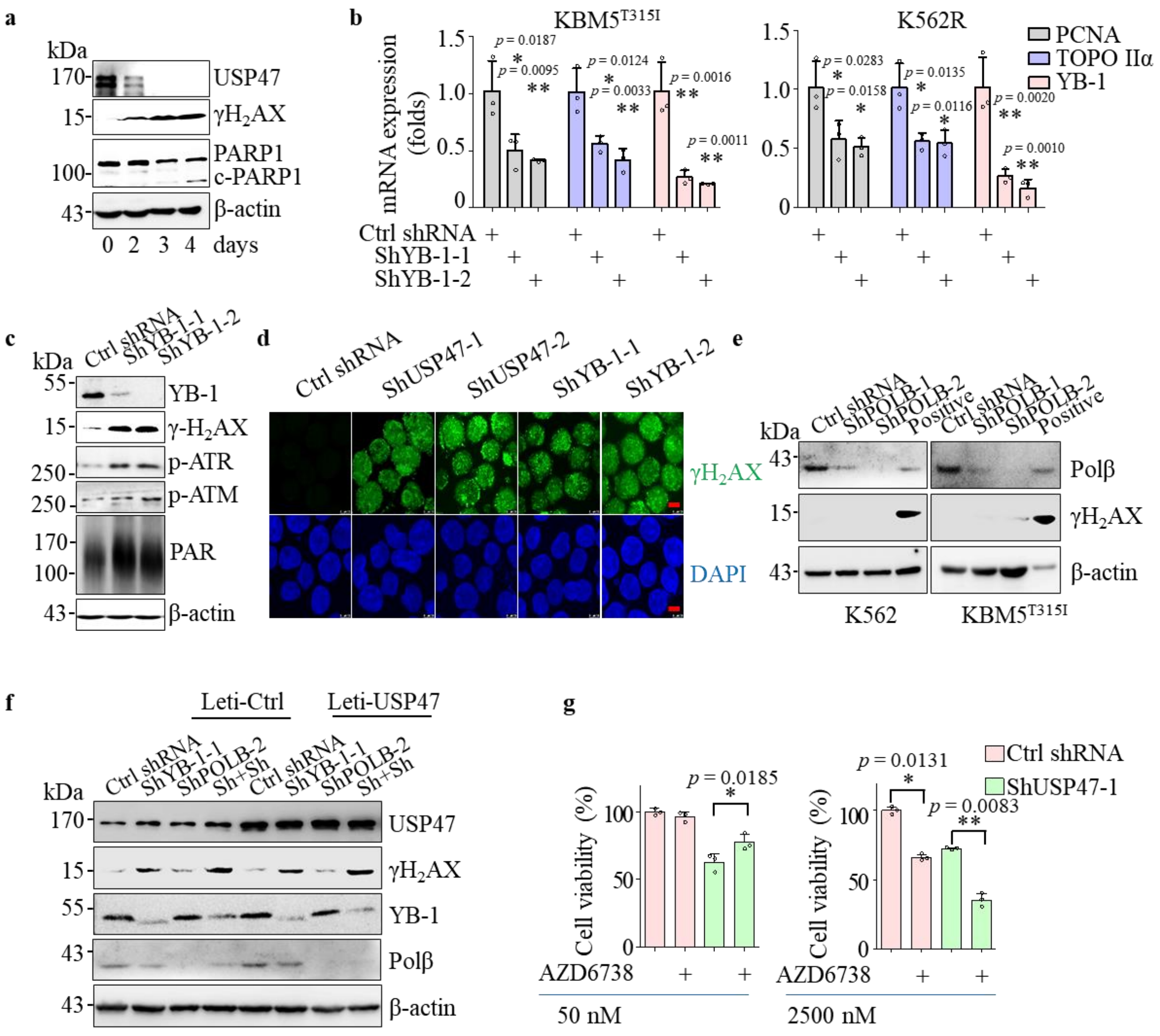

Supplementary Fig. 6 YB-1 and USP47 regulate DNA damage repair in CML cells. a Time course analysis of the USP47 knockdown-induced DNA damage response by Western blot in KBM5^{T315I} cells. **b** *YB-1*, *PCNA*, and *TOPO IIα* mRNA levels after *YB-1* knockdown at day 7 in KBM5^{T315I} and K562R cells (n=3 biologically independent samples per group). Data are mean ± s.d., *P* values were analyzed by one-way analysis of variance (ANOVA). *, *p*<0.05; **, *p*<0.01. **c** DNA damage protein expression was measured by Western blot after YB-1 knockdown in the KBM5^{T315I} cells. **d** In USP47 or YB-1 silenced KBM5^{T315I} cells, γH_2AX foci were detected by immunofluorescence staining. Scale bars = 7.5 µm. **e** γH_2AX expression was measured by Western blot after POLB knockdown in the K562 and

KBM5^{T315I} cells. **f** Overexpress USP47 in YB-1 and/or POLB depleted K562 cells, then the indicated proteins were detected by Western blot. **g** After transfected with USP47 specific shRNA or control shRNA in K562 cells for 48 hours, then the cells were treated with AZD6738 (50 nM or 2500 nM) for 12 hours. Cell viability was measured by CCK-8. (n=3 biologically independent samples per group) Data are mean ± s.d., *P* values were analyzed by one-way analysis of variance (ANOVA). *, $p<0.05$; **, $p<0.01$. Source data are provided as a Source Data file.

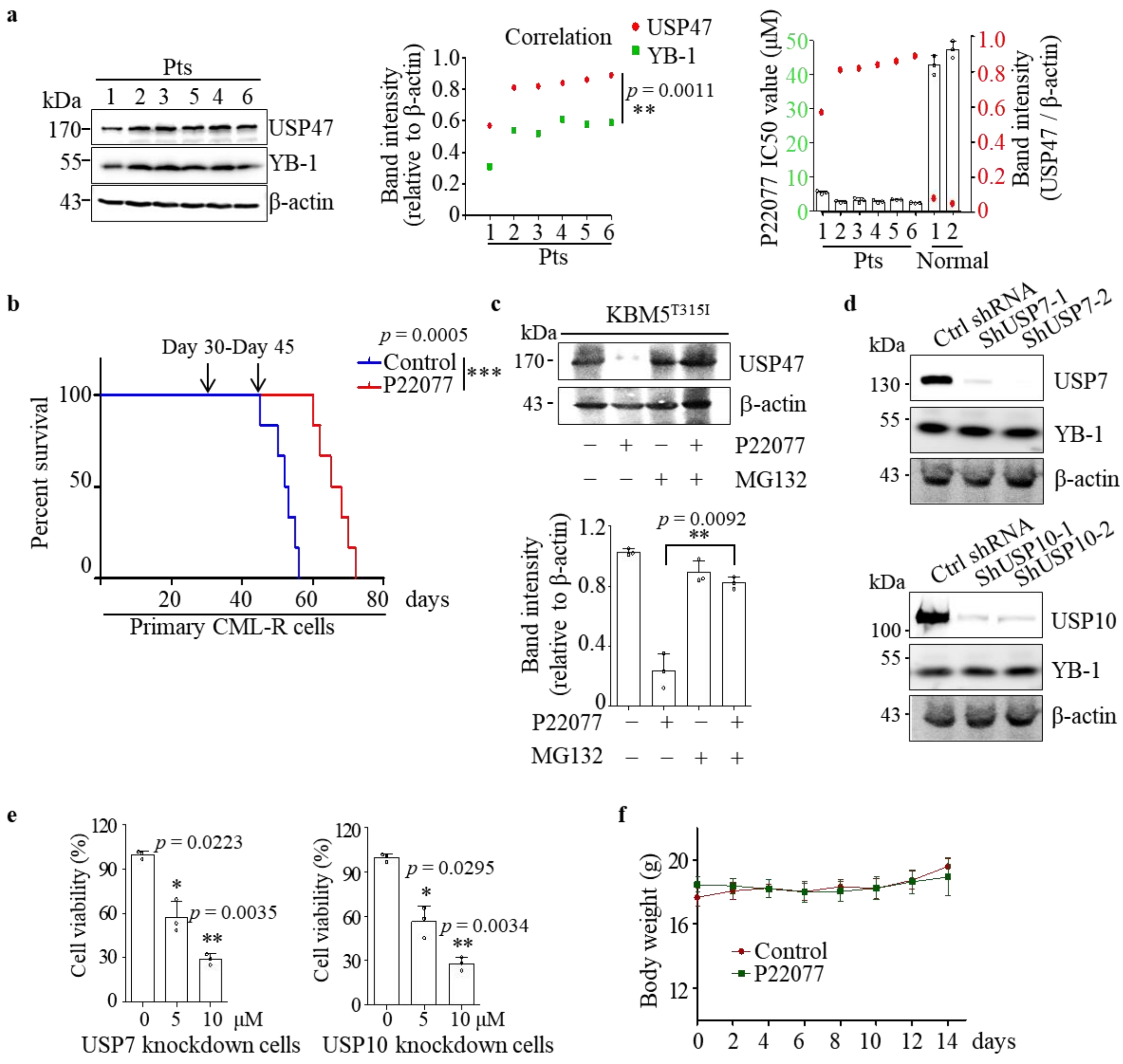

Supplementary Fig. 7 P22077 targets USP47 and shows toxic effects on CML cells. a Protein level of USP47 and YB-1 in primary CML cells (left) and their correlations were shown (middle). Primary CML cells were treated with P22077. The IC50 value was measured. The correlation between USP47 level and IC50 to P22077 was shown (right) (n=3 biologically independent samples per group). **, *p*<0.01. *P* value was analyzed by Pearson's r correlation test, r=0.9733, r^2=0.9473. **b** P22077 (30 mg/kg/day) or vehicle were given to mice transplanted with primary CML-R cells by intraperitoneal injection (n=6 biologically independent samples per group) from day 30 to day 45. ***, *p*<0.001. *P* value was analyzed by Mantel–Cox-log-rank test. **c** KBM5^{T315I} cells were treated with P22077 (10 μM) with or without MG132 (10 μM) for 8 hours, then proteins were extracted and subjected to Western

blot. The band intensity is shown by histogram (n=3 biologically independent experiments). Data are presented as mean ± s.d. *P* values were analyzed by one-way analysis of variance (ANOVA), **, *p*<0.01. **d** USP7 or USP10 was silenced in $KBM5^{T315I}$ cells with a retroviral transduction system, and the expression of YB-1 protein was examined by Western blot. **e** USP7 or USP10 stably-knockdown K562 cells were treated with different concentrations of P22077 for 48 hours, the cell viability was measured by CCK8 assay (n=3 biologically independent samples per group). Data are mean ± s.d. *P* values were analyzed by one-way analysis of variance (ANOVA). *, *p*<0.05, **, *p*<0.01. **f** Wild-type female C57BL/6 mice (6 weeks, n=5 biologically independent samples per group) were treated with P22077 (30 mg/kg) or control solvent for 14 days. The mice were weighed every two days. Source data are provided as a Source Data file.

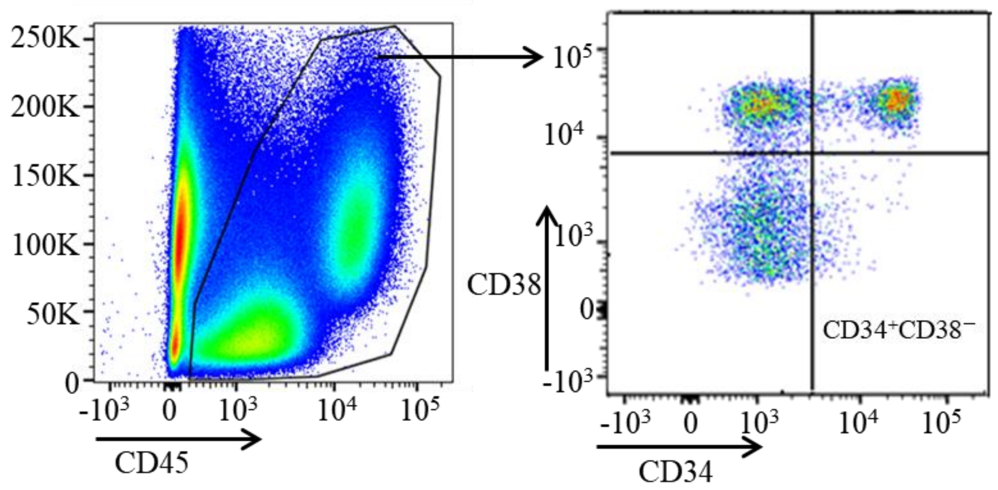

Supplementary Fig. 8 Gating strategy for human leukemia stem/progenitor cells ($CD45^+CD34^+CD38^-$) in the BM of B-NDG mice. The gating strategy corresponds to Figure 7i.

Supplementary Table 1 The information of CML patients

ID	Age (years)	Gender (F/M)	Imatinib-resistant	2nd TKIs-resistant
CML-1	52	F	Yes	No
CML-2	60	M	No	No
CML-3	68	M	Yes	No
CML-4	58	M	No	No
CML-5	60	M	No	No
CML-6	57	M	No	No
CML-7	53	F	No	No
CML-8	57	F	No	No
CML-9	59	M	No	No
CML-10	55	M	No	No
CML-11	60	M	Yes	Yes
CML-12	61	M	No	No
CML-13	56	F	No	No
CML-14	57	F	No	No
CML-15	53	M	No	No
CML-16	58	M	Yes	Yes
CML-17	67	M	No	No
CML-18	65	M	No	No
CML-19	65	M	No	No
CML-20	70	M	Yes	No
CML-21	66	M	No	No
CML-22	71	M	No	No
CML-23	70	M	No	No
CML-24	63	M	Yes	Yes
CML-25	59	M	No	No
CML-26	56	F	No	No
CML-27	63	F	Yes	No
CML-28	73	M	No	No
CML-29	68	M	No	No
CML-30	73	M	No	No
CML-31	61	F	Yes	No
CML-32	63	M	No	No
CML-33	59	M	No	No
CML-34	57	F	No	No
CML-35	54	M	Yes	No
CML-36	65	F	Yes	No
CML-37	57	M	No	No
CML-38	64	M	No	No
CML-39	65	F	No	No
CML-40	68	M	No	No
CML-41	73	M	No	No

Supplementary Table 2. ShRNA target sequences

**USP47 shRNA**	
USP47-shRNA-1	GAATCTGTCTTGAAACCAA
USP47-shRNA-2	GCAATGACTTGCTATTTGAA
**BCR-ABL shRNA**	
BCR-ABL-shRNA	AGCAGATCGAGACCATCTT
**YB-1 shRNA**	
YB-1-shRNA-1	GGTTCCCACCTTACTACAT
YB-1-shRNA-2	GGTCATCGCAACGAAGGTT
**USP7 shRNA**	
USP7-shRNA-1	TGCGAAATCTGCCATGGAA
USP7-shRNA-2	CTCAGAACCCTGTGATCAA
**USP10 shRNA**	
USP10-shRNA-1	CTCTCTTTAGTGGCTCTTT
USP10-shRNA-2	CCTATGTGGAAACTAAGTA
**STAT5 shRNA**	
STAT5-shRNA-1	GCCATGAATCGCTTCTCTTT
STAT5-shRNA-2	GCCATGACCTACTCATTAAC
**POLB shRNA**	
POLB shRNA-1	TACCCACACAAAATAAAGAG
POLB shRNA-2	CTGATATCAATTCTTCTGTG

Supplementary Table 3. Real-time PCR primer sequences for DUBs

Name	Forward (5’-3’)	Reverse (5’-3’)
USP1	AACTGCCATCATTATACTG	TTGTGCTCCATTCTTCTA
USP2	TGCTGAGACCCGACATCACT	TGGGGTCTATCCGGTAGCTA
USP3	CTGTTATCGCTGTGATGAT	CCAAGTTCTGTAAGTGTTCT
USP4	GTCTTGTGAACTCCTATG	CAGATTCTTGCTCATCAT
USP5	TGGCTATTGGTGTTGAAG	GGCAATCTCCAGGTAATC
USP6	ATGTGGAACTGAGAAGAA	GCAATGATGTAACCTGTAA
USP7	GCAAGTGCAGATAGTCGCAGAGGAC	CCATGGTCTGAGAGAGGCTCTGAAC
USP8	ACTATACACAATGATGACGGATA	TGGACTGATGGCTTCTTC
USP9X	AGGTGGTGGAATGCTTAT	GAGGTCTGGTGGTGATAG
USP9Y	CTCAGTATCAACAGAATAATC	TCTTCATTGCCTTCATAA
USP10	GAATCTGTCCAAGGTTATAC	CCACCAGTCTTCTCATAA
USP11	AGTAGAACTGCTGCTTGT	ATAGAATCGGTATGGCTGAA
USP12	TGCGTATAAGAGTCAACCT	GAGTGGCTATGCTATGGA
USP13	ACGAGCAACGAATAATAAC	TTCTCCATCTCAATCACAA
USP14	AAATGGCTTCAGCGCAGTAT	TTCACCTTTCTCGGCAAACT
USP15	GACAGGTATTAGTGATAGA	GATAGAGATGAAGGAGAG
USP16	AGAACCTGAGTTAAGTGATG	CAATCTTGCTGTCCTCTC
USP17L30	AACAAGATTGCCAAGAATG	TAGAGGACATAGACGAGAG
USP18	AATGGTTCTGCTTCAATG	CCAGTGGTAGTTAGGATT
USP19	TGGACCTGAGCAAGTTCT	ATAGTGGTTGATGACAGCATATAG
USP20	TATGTTGGCTGCGGAGAA	AGGTTCACGGTCAAGTTGT
USP21	AGAACCTGAGTTAAGTGATG	CAATCTTGCTGTCCTCTC
USP22	GAGAGCAGGATGAATGGA	AACAGCAAACAGGGAATAC
USP24	TGAGATGCCAGTTATTAGA	AGTTATCCAGCCAAGTAA
USP25	TCTCCTGTTGACGATATTG	TTCTGTTGTGCTTGGTAA
USP27X	ATGTGTAAGGACTATGTATATGAC	GAGGTGGAGGCTTGTAAT
USP28	AATGCTGGACACTATTGG	GGAAGATTCAGTAACAGAGAT
USP29	ATGCTGTTCTCAAGGTAG	AACTCTTCTGCTTACACAT
USP30	ACTGATGATGAGGTCTTAG	TTCCAATGACGAGGTAAT
USP32	ATGTGGAACTGAGAAGAA	GCAATGATGTAACCTGTAA
USP34	GTTGTTGACTATGCTAAT	ACTGAGAAGGATTGTATT

USP35	ATTAGCAGGATGATTGAC	TGAACTTCTTAACAGCAG
USP37	CATCAGTGTTGTCAGTCA	CTCCAGGTCATTGTAAGTAA
USP38	CAGCATATTCCTCTTCAG	ATAGCCAGTCAATCATTC
USP39	GGCATCACTGAGAAGGAA	AAGATTAGATATGGAGGCAACT
USP40	CTCTTCTCAGTTATTATACAC	CAATCTCTTCTTCACTCT
USP41	AATGGTTCTGCTTCAATG	CCAGTGGTAGTTAGGATT
USP42	ATCAGTATTCACAACAAG	ATTGGTAATGGTAGAAGA
USP43	TGGTACAGTTATGATGAC	TGATAGAACAGGATATAAGC
USP44	ACCAGTCACAGATACAGTAG	TTGATGCTCCACCACTTA
USP45	CTGCTCTGCTACTTGTAA	GCCACTATGTTCCACTAT
USP46	CGATGCTTGAACTGTGAA	TGTGTTGCTGAAGTCTCT
USP47	GCTAATGGACTTGACTCT	CACTCTCATCATATTCACTATC
USP48	CACTCTACTTATGTCCAA	ATCAATGTATCGCCTATT
USP49	GCTTGTGACCAGTGTAAC	GAGGTAGTCTGTAGATCATTAAC
USP50	GGATTACCACTGAGACAT	AGACTTCGTTCTTGTAGG
USP51	AAGACAAGCAATCAACCT	CCTGGACAATACAATTCATAA
USP52	AGGTGGTGGATTACTTGAC	GTTAGGTGCTTGGAGGAA
USP53	GCTCCTCAACTAACGATT	CTCATTTGGACAGGTAGAA
USP54	ATTCTTCTCTTGCCTTAA	CTTCTCTTGGTATATGTCT
CYLD	AGGCTTGGAGATAATGATTGG	GCAGAATAAGGTTGAGTCTAAGTA
USPL1	CTCCACATAAGCCTCAGAA	TCCACCGTCAATAGCAATA
UCHL1	CAAGAAGTTAGTCCTAAAGTGTA	GCGTGAATAAGTCCGATT
UCHL3	GGCAATTCGTTGATGTAT	TCTTCCTCTTCTGTTCTG
UCHL5	TTAATAATGCTTGTGCTACTC	CTGATAATGTCTCGCCTAA
BAP1	TATCTTCCTGTTCAAATG	CAATATCATCATCAATCAC
ATXN3	AACATTGCCTGAATAACT	TAGTAACTCCTCCTTCTG
ATXN3L	ACCAATAGAGAAGATGAACA	GAAGCAGGAGTTACACAT
OTUB1	AGGAGTATGCTGAAGATGAC	CTTGCGGATGTACGAGTA
OTUB2	GAGGAGCACAAGTTCAGAA	ACACTGAGCCATCCTTCT
OTUD1	AGATGCTGAATGTGAATA	TAATGAATCATGGTAGACA
OTUD3	TCTGAAGACGACCTGAGAG	CGAAGCACGGCAATTATTG
OTUD4	ATCCAAGCAGTTCTATAATCA	ACTCCTCACTTCTCACAT
HIN1L	GAAGCGATTATAGGAGGAT	AGTTACTTGTGAAGGAGAA
OTUD5	GGACTATCTGATGAAGAATGC	TCCGCTTCCTGTTAATGTA

OTUD6A	AGTAGCATTGAATCTGTC	ACTCCATTCTTTCTCTCT
OTUD6B	CAAGAATGACAAGAAGAG	TAACAGCAACAGAATCTA
YOD1	ATACACAGACAGTAAGAA	TATCATCATTAGAGGAGAA
A20	AATGAGATGAAGGAGAAG	ATTGATGAGATGAGTTGT
OTUD7A	AGCAATTCTAACAGCAATAAC	GTCTTGTCCTTCTCCTTG
OTUD7B	AGCAGACACAGCAGAATA	TCAGTTCATTCCACTCCTT
TRABID	GACTGATTGGCTCTTCCT	TGATGACTTGTATGCTTCTATG
VCPIP1	TGGAGTAGTAACAATGAGA	TGAAGCCTGAATAGAAGA
BRCC3	GATTACTATGGTCACTTG	CATCATCCTCATCAATAG
COPS5	ACTCAGATGCTCAATCAG	TGCGGATATTGTTCTTGT
COPS6	CCCTCTTTCTGAAGTTGA	GCCTCTCCATTGATTATATC
PSMD14	CAATGCTAATATGATGGTCTTA	GTAATGGAGTAATAATGTCTGTT
PSMD7	AAGAATAGTTGGCTGGTA	CGGAATTAGGACAGTATCT
AMSH	GAGTTGAGATTATCCGAATG	AGAGCGTGATATACTTGTT
AMSH-LP	ATGGAGAATGTAGAGGAAT	TTGATAGGAACAGTGAGT
MPND	GCAGCCATCAACAAGTTC	TCAGGTGACTGTGGAAGT
MYSM1	ATTGTATTGGACGGATTC	GTTGGTATGCTTCTACTG
PRPF8	GGATGAAGACTGGAATGAAT	TGGTGTGGAAGATTGTTG
EIF3F	TTCCTGATGAGCCTGGTTA	GGTCATTGATGTTGCTGTTG
EIF3H	GATGGACAGAGTGGATGAA	GACGCTGCTGATACTGAT
JOSD1	GTGGATTGGAGGCGAGAG	AGCAGGAGTTCACAGTTCTT
JOSD2	TGAGATCTGCAAGAGGTT	ATCACATTGACATCATAGTTG
JOSD3	TTGACAGTCGTAGATACA	TTCAGTAATATCCTCTTCTTC

Supplementary Table 4. Real-time PCR primer sequences

Name	Forward (5'-3')	Reverse (5'-3')
BCR-ABL	CGGGAGCAGCAGAAGAAGTGT	CGAAAAGGTTGGGGTCATTTTC
hUSP47	GCTAATGGACTTGACTCT	CACTCTCATCATATTCACTATC
hYB-1	AAGTGATGGAGGGTGCTGAC	TTCTTCATTGCCGTCCTCTC
hPOLB	GTATTACTGTGGTGTTCTCTATT	TGGTGTACTCATTGATTGTG
hGAPDH	CTTAGCACCCCTGGCCAAG	TGGTCATGAGTCCTTCCACG
mUsp1	GGAACTACGACGATGAAG	CACCGAGAAGTCCAATAC
mUsp2	AGAGACCTGGACTTGAGA	TGATTGGACACAGCATACA
mUsp3	TATTCCAAGTCAGTTCAG	TCTCATCAAGTTCCTCTA
mUsp4	TTGAAGGAGCACTTAATCG	ACACAGCCATACCAATTC
mUsp6	GTCCTTACTGCTATGTCT	CTTGAAGTTGCTCTATTACA
mUsp7	TGATGATGATCTGTCTGT	CAACTGCTGAGGAATATC
mUsp8	CAAGCAACAGCAGGATTA	ACTTCAGCCTCTTCGTAT
mUsp9x	GAGAGGATGGCTGAATGGAT	ACTGTGGTTGATGAAGGCTAT
mUsp10	GATGGAAGTCAAAGAAGG	CACTGGCTTATGTATTAGG
mUsp14	TGGCTACTATGACTTACAA	GTTACAATGCTGACCTTAT
mUsp19	AAGAGGAAGAGAAGAAGG	AAGACTGAATGACGCTAT
mUsp36	GTGTGCTAAGTGTAAGAAGA	AGAGTCAGGACATTGGAT
mUsp47	TATCACAAGTAGTAGAAGAAC	ACTCTCGTCATATTCACT
mGAPDH	CTTAGCCCCCCTGGCCAAG	TGGTCATGAGCCCTTCCACA

h=human, m=mouse
