# Supplementary material for: Targeting USP47 overcomes tyrosine kinase inhibitor resistance and eradicates leukemia stem/progenitor cells in chronic myelogenous leukemia
Source: Nat Commun. 2021 Jan 4;12:51. doi: 10.1038/s41467-020-20259-0 (PMC7782553; doi:10.1038/s41467-020-20259-0)
Supplement: Supplementary file 3 — Reporting Summary [file 41467_2020_20259_MOESM3_ESM.pdf]

nature research

Corresponding author(s): Yingli Wu

Last updated by author(s): Nov 3, 2020

Reporting Summary

Nature Research wishes to improve the reproducibility of the work that we publish. This form provides structure for consistency and transparency in reporting. For further information on Nature Research policies, see Authors & Referees and the Editorial Policy Checklist .

Statistics

For all statistical analyses, confirm that the following items are present in the figure legend, table legend, main text, or Methods section.

n/a	Confirmed	
[ ]	[x]	The exact sample size (*n*) for each experimental group/condition, given as a discrete number and unit of measurement
[ ]	[x]	A statement on whether measurements were taken from distinct samples or whether the same sample was measured repeatedly
[ ]	[x]	The statistical test(s) used AND whether they are one- or two-sided<br>*Only common tests should be described solely by name; describe more complex techniques in the Methods section.*
[x]	[ ]	A description of all covariates tested
[x]	[ ]	A description of any assumptions or corrections, such as tests of normality and adjustment for multiple comparisons
[ ]	[x]	A full description of the statistical parameters including central tendency (e.g. means) or other basic estimates (e.g. regression coefficient) AND variation (e.g. standard deviation) or associated estimates of uncertainty (e.g. confidence intervals)
[ ]	[x]	For null hypothesis testing, the test statistic (e.g. *F*, *t*, *r*) with confidence intervals, effect sizes, degrees of freedom and *P* value noted<br>*Give P values as exact values whenever suitable.*
[x]	[ ]	For Bayesian analysis, information on the choice of priors and Markov chain Monte Carlo settings
[x]	[ ]	For hierarchical and complex designs, identification of the appropriate level for tests and full reporting of outcomes
[ ]	[x]	Estimates of effect sizes (e.g. Cohen's *d*, Pearson's *r*), indicating how they were calculated

Our web collection on statistics for biologists contains articles on many of the points above.

Software and code

Policy information about availability of computer code

Data collection	Western blot data were collected by LAS-4000 (GE health care).<br>H&E and IHC image data were collected by CKX31 (Olympus).<br>Real-time PCR data were collected by ABI7900 (ABI).<br>Flow cytometry data were collected by BD FACS Calibur and BD LSRFortessa(BD).<br>IF imaging data were collected by A1R-si (Nikon).<br>All mass spectrometric experiments are performed on a LTQ orbitrap “XL” mass spectrometer connected to an Easy-nLC 1000 via an Easy Spray (Thermo Fisher Scientific).
Data analysis	Software Flowjo version 10 were used for FACS analyses.<br>Prism software (GraphPad, version 6.02) was used for statistical analyses.<br>Image J software (version 1.46) was used for quantification of immunoblot intensity.<br>GraphPad Prism 6 was used to draw graphs in the study. Microsoft PowerPoint 2010 and Photoshop CS3 (version 6.2) was used to crop images from unprocessed images.<br>All MS/MS spectrum were analyzed by Mascot (Matrix Science, London, UK; version 2.4.1) searching against the uniprot human database. MS/MS-based peptide and protein identifications were validated by Scaffold (version Scaffold 4.2.1, Proteome Software Inc., Portland, OR) using the Scaffold Local FDR algorithm.

For manuscripts utilizing custom algorithms or software that are central to the research but not yet described in published literature, software must be made available to editors/reviewers. We strongly encourage code deposition in a community repository (e.g. GitHub). See the Nature Research guidelines for submitting code & software for further information.

Data

Policy information about availability of data

All manuscripts must include a data availability statement. This statement should provide the following information, where applicable:

- Accession codes, unique identifiers, or web links for publicly available datasets
- A list of figures that have associated raw data
- A description of any restrictions on data availability

Data supporting the findings of this study are available within the article and its Supplementary Information files or from the corresponding author upon reasonable request. The source data underlying Figs. 1a-h, 2a-f, 3b-e, 3i,4a, 4c, 4g, 4h, 5b-l, 6a-c, 6e-m, 7a-l and supplementary Figs. 1a-c, 2b-c, 3a-c, 4a-c, 5a-d, 6a-c, 6e-g, 7a-f are provided as a Source Data file.

Field-specific reporting

Please select the one below that is the best fit for your research. If you are not sure, read the appropriate sections before making your selection.

- [x] Life sciences
- [ ] Behavioural & social sciences
- [ ] Ecological, evolutionary & environmental sciences

For a reference copy of the document with all sections, see nature.com/documents/nr-reporting-summary-flat.pdf

Life sciences study design

All studies must disclose on these points even when the disclosure is negative.

Sample size	For cell based experiments, samples are triplicated and more than three independent experiments were performed. For mice experiments, required sample sizes were estimated based on our experience performing similar experiments in previous publications. Sample sizes are stated in figure legends.
Data exclusions	No data were excluded from the analyses.
Replication	Experiments were performed in three independent biological replicates. All attempts were successful at replication although there were some variations of values.
Randomization	B-NDG Mice, recipient mice for transplantation and in vitro experimental samples were allocated randomly.
Blinding	For cell based experiments, investigators were blinded during data collection and analysis. For mice experiments, investigators were blinded during data collection and analysis. However, for gene knockout mice experiments, investigators were partial blinded for choosing mice because of mice genotype. For mice experiments with drug treatment, investigators were partial blinded due to the weak yellow solution of P22077.

Reporting for specific materials, systems and methods

We require information from authors about some types of materials, experimental systems and methods used in many studies. Here, indicate whether each material, system or method listed is relevant to your study. If you are not sure if a list item applies to your research, read the appropriate section before selecting a response.

Materials & experimental systems

n/a	Involved in the study	
[ ]	[x]	Antibodies
[ ]	[x]	Eukaryotic cell lines
[x]	[ ]	Palaeontology
[ ]	[x]	Animals and other organisms
[ ]	[x]	Human research participants
[x]	[ ]	Clinical data

Methods

n/a	Involved in the study	
[x]	[ ]	ChIP-seq
[ ]	[x]	Flow cytometry
[x]	[ ]	MRI-based neuroimaging

Antibodies

Antibodies used	p-Bcr antibody , cell signaling technology, Cat#3901, Lot#1, Dilution: 1:1000(WB)<br>c-Abl antibody, cell signaling technology, Cat#2862, Lot#13, Dilution: 1:1000(WB)<br>p-CrkL antibody, cell signaling technology, Cat#3181, Lot#7, Dilution: 1:1000(WB)<br>CrkL antibody, cell signaling technology, Cat#3182, Lot#5, Dilution: 1:1000(WB)<br>p-ERK antibody, cell signaling technology, Cat#9101, Lot#26, Dilution: 1:1000(WB)<br>ERK antibody, cell signaling technology, Cat#9102, Lot#25, Dilution: 1:1000(WB)

p-P38 MAPK antibody, cell signaling technology, Cat#9215, Lot#19, Dilution: 1:1000(WB)
p-JNK MAPK antibody, cell signaling technology, Cat#9251, Lot#10, Dilution: 1:1000(WB)
p-AKT antibody, cell signaling technology, Cat#4060, Lot#30, Dilution: 1:1000(WB)
caspase-3 antibody, cell signaling technology, Cat#9661,Lot#45,Dilution: 1:1000(WB)
γH2AX antibody, cell signaling technology, Cat#9718, Lot#1, Dilution: 1:1000(WB),1:200(IHC), 1:100(IF)
p-p53 antibody, cell signaling technology, Cat#9284, Dilution:1:1000(WB)
p-ATR antibody, cell signaling technology, Cat#2853, Lot#9, Dilution:1:1000(WB)
p-ATM antibody, cell signaling technology, Cat#4526, Lot#14,Dilution:1:1000(WB)
PAR antibody, cell signaling technology, Cat#83732, Dilution:1:1000(WB)
β-catenin antibody, cell signaling technology, Cat#9587, Lot#2, Dilution:1:1000(WB)
USP47 antibody, Santa Cruz Biotechnology, Cat#sc-100633, Lot#J1314, Dilution: 1:200(IHC), 1:500(WB)
STAT5 antibody, Santa Cruz Biotechnology, Cat#sc-74442, Dilution: 1:500(WB)
USP14 antibody, Santa Cruz Biotechnology, Cat#sc-100630, Lot#J3013, Dilution: 1:500(WB)
Ubiquitin antibody, Santa Cruz Biotechnology, Cat#sc-8017, Lot#H0409, Dilution: 1:500(WB)
GFP antibody, Santa Cruz Biotechnology, Cat#sc-9996, Lot#J0813, Dilution: 1:500(WB),1:100(IHC)
PCNA antibody, Santa Cruz Biotechnology, Cat#sc-53407, Dilution: 1:200(IHC)
PARP1 antibody, Santa Cruz Biotechnology, Cat#sc-56197, Dilution: 1:500(WB)
p53 antibody, Santa Cruz Biotechnology, Cat#sc-126, Dilution: 1:500(WB)
Polβ antibody, Proteintech, Cat#18003-1-AP, Lot#00009472, Dilution: 1:1000(WB),1:200(IHC)
YB-1 antibody, Proteintech, Cat#20339-1-AP, Lot#00046294, Dilution: 1:1000 (WB),1:200(IHC)
Flag tag antibody, Proteintech, Cat#66008-2-Ig, Dilution: 1:1000(WB)
USP10 antibody, Proteintech, Cat#19374-1-AP, Lot#00015015, Dilution: 1:1000(WB)
TOPOIIα antibody, Proteintech, Cat#20233-1-AP, Dilution: 1:200(IHC)
SIRT6 antibody, Proteintech, Cat#13572-1-AP, Lot#00004562, Dilution: 1:1000(WB)
HRP-β-actin antibody, Proteintech, Cat#HRP-66009,Lot#21000002 Dilution: 1:5000(WB)
USP7 antibody, Bethyl Laboratories Inc, Cat#A300-033A, Dilution: 1:1000(WB)
USP16 antibody, GeneTex Inc., Cat#GTX16439, Lot#39631, Dilution: 1:1000(WB)
Lineage Antibody Cocktail, ebioscience Inc., Cat#88-7772-72, Dilution: 1:200(F)
sca-1 antibody, ebioscience Inc., Cat#25-5981-82, Dilution: 1:200(F)
c-kit antibody, ebioscience Inc., Cat#17-1171-82, Dilution: 1:200(F)
CD34 antibody, ebioscience Inc., Cat#11-0349-42, Dilution:1:200(F)
CD38 antibody, ebioscience Inc., Cat#12-0389-42, Dilution:1:200(F)
CD45 antibody, ebioscience Inc., Cat#17-0459-42, Dilution:1:200(F)
Gr-1 antibody, Miltenyi Biotec., Cat#130-119-794, Dilution: 1:200(F)
CD45 antibody,ServiceBio.,Cat#GB14038,Dilution: 1:200(IHC)

Validation

p-Bcr antibody https://www.cellsignal.cn/products/primary-antibodies/phospho-bcr-tyr177-antibody/3901?N=4294956287&Ntt=3901&fromPage=plp
c-Abl antibody, https://www.cellsignal.cn/products/primary-antibodies/c-abl-antibody/2862?N=4294956287&Ntt=2862&fromPage=plp
p-CrkL antibody, https://www.cellsignal.cn/products/primary-antibodies/phospho-crkl-tyr207-antibody/3181?N=4294956287&Ntt=3181&fromPage=plp
CrkL antibody, https://www.cellsignal.cn/products/primary-antibodies/crkl-32h4-mouse-mab/3182?N=4294956287&Ntt=3182&fromPage=plp
p-ERK antibody, https://www.cellsignal.cn/products/primary-antibodies/phospho-p44-42-mapk-erk1-2-thr202-tyr204-antibody/9101?N=4294956287&Ntt=9101&fromPage=plp
ERK antibody, https://www.cellsignal.cn/products/primary-antibodies/p44-42-mapk-erk1-2-antibody/9102?N=4294956287&Ntt=9102&fromPage=plp
p-P38 MAPK antibody, https://www.cellsignal.cn/products/primary-antibodies/phospho-p38-mapk-thr180-tyr182-3d7-rabbit-mab/9215?N=4294956287&Ntt=9215&fromPage=plp
p-JNK MAPK antibody, https://www.cellsignal.cn/products/primary-antibodies/phospho-sapk-jnk-thr183-tyr185-antibody/9251?N=4294956287&Ntt=9251&fromPage=plp
p-AKT antibody, https://www.cellsignal.cn/products/primary-antibodies/phospho-akt-ser473-d9e-xp-rabbit-mab/4060?N=4294956287&Ntt=4060&fromPage=plp
caspase-3 antibody, https://www.cellsignal.cn/products/primary-antibodies/cleaved-caspase-3-asp175-antibody/9661?N=4294956287&Ntt=9661&fromPage=plp
γH2AX antibody, https://www.cellsignal.cn/products/primary-antibodies/phospho-histone-h2a-x-ser139-20e3-rabbit-mab/9718?N=4294956287&Ntt=9718&fromPage=plp
p-p53 antibody, https://www.cellsignal.cn/products/primary-antibodies/phospho-p53-ser15-antibody/9284?N=4294956287&Ntt=9284&fromPage=plp
p-ATR antibody, https://www.cellsignal.cn/products/primary-antibodies/phospho-atr-ser428-antibody/2853?N=4294956287&Ntt=2853&fromPage=plp
p-ATM antibody, https://www.cellsignal.cn/products/primary-antibodies/phospho-atm-ser1981-10h11-e12-mouse-mab/4526?N=4294956287&Ntt=4526&fromPage=plp
PAR antibody, https://www.cellsignal.cn/products/primary-antibodies/poly-mono-adp-ribose-e6f6a-rabbit-mab/83732?N=4294956287&Ntt=83732&fromPage=plp
β-catenin antibody, https://www.cellsignal.cn/products/primary-antibodies/b-catenin-antibody-carboxy-terminal-antigen/9587?

N=4294956287&Ntt=9587&fromPage=plp
USP47 antibody, https://www.scbt.com/zh/p/usp47-antibody-4e7?requestFrom=search, IHC validation by ourself.
STAT5 antibody, https://www.scbt.com/zh/p/stat5-antibody-a-9?requestFrom=search
USP14 antibody, https://www.scbt.com/p/usp14-antibody-6e6?requestFrom=search
Ubiquitin antibody, https://www.scbt.com/zh/p/ubiquitin-antibody-p4d1?requestFrom=search
GFP antibody, https://www.scbt.com/p/gfp-antibody-b-2?requestFrom=search
PCNA antibody, https://www.scbt.com/p/pcna-antibody-pc11?requestFrom=search
PARP1 antibody, https://www.scbt.com/zh/p/parp-1-antibody-5a5
p53 antibody, https://www.scbt.com/zh/p/p53-antibody-do-1?requestFrom=search
Polβ antibody, https://www.ptglab.com/products/POLB-Antibody-18003-1-AP.htm
YB-1 antibody, https://www.ptglab.com/products/YBX1-Antibody-20339-1-AP.htm
Flag tag antibody, https://www.ptglab.com/products/Flag-tag-Antibody-66008-2-Ig.htm
USP10 antibody, https://www.ptglab.com/products/USP10-Antibody-19374-1-AP.htm
TOPOIIα antibody, https://www.ptglab.com/products/TOP2A-Specific-Antibody-20233-1-AP.htm
SIRT6 antibody, https://www.ptglab.com/products/SIRT6-Antibody-13572-1-AP.htm
HRP-β-actin antibody, https://www.ptglab.com/products/beta-Actin-Antibody-HRP-66009.htm
USP7 antibody, https://www.bethyl.com/product/A300-033A?referrer=search
USP16 antibody, https://www.genetex.cn/Product/Detail/USP16-antibody/GTX16439#datasheet
Lineage Antibody Cocktail, https://www.thermofisher.com/cn/zh/antibody/product/Mouse-Hematopoietic-Lineage-Antibody-Cocktail/88-7772-72
sca-1 antibody, https://www.thermofisher.com/cn/zh/antibody/product/Ly-6A-E-Sca-1-Antibody-clone-D7-Monoclonal/25-5981-82
c-kit antibody, https://www.thermofisher.com/cn/zh/antibody/product/CD117-c-Kit-Antibody-clone-2B8-Monoclonal/17-1171-82
CD34 antibody, https://www.thermofisher.com/cn/zh/antibody/product/CD34-Antibody-clone-4H11-Monoclonal/11-0349-42
CD38 antibody, https://www.thermofisher.com/cn/zh/antibody/product/CD38-Antibody-clone-HIT2-Monoclonal/12-0389-42
CD45 antibody, https://www.thermofisher.com/cn/zh/antibody/product/CD45-Antibody-clone-HI30-Monoclonal/17-0459-42
Gr-1 antibody, https://www.miltenyibiotec.com/CN-en/products/gr-1-antibody-anti-mouse-rb6-8c5.html#percp:30-ug-in-200-ul
CD45 antibody, https://www.servicebio.cn/goodsdetail?id=301

Eukaryotic cell lines

Policy information about cell lines

Cell line source(s)	K562, K562R, 32D, HEK293T cells were obtained from ATCC, KBM5T315I cells, originally established by Clara Ricci (Cancer Research, 2002,62,5995-5998), were kindly provided by Dr Jingxuan Pan (sun yat-sen university, China).
Authentication	Cells were authenticated by examination of morphology and growth characteristics.
Mycoplasma contamination	Cells were determined to be mycoplasma negative.
Commonly misidentified lines (See ICLAC register)	None.

Animals and other organisms

Policy information about studies involving animals; ARRIVE guidelines recommended for reporting animal research

Laboratory animals	C57BL/6-Usp47 knockout mice were obtained from MMRRC (Mutant Mouse Resource & Research Centers, USA). BALB/c-Usp47 knockout mice were generated by backcrossing BALB/c background and C57BL/6 Usp47 knockout mice for over 10 generations. B-NDG mice were obtained from Jiangsu Biocytogen Co., Ltd. (Nantong, China). The mice were bred in equipped animal facility with temperature at 20~25 °C and humidity at 30~70%, with a 12 h light-dark cycle and ad libitum access to regular chow diet and water. The 6-8 week old female mice were used for experiments.
Wild animals	The study did not involve wild animals.
Field-collected samples	This study did not use field-collected samples.
Ethics oversight	Animal care and experiments were compliant with all of the relevant ethical regulations regarding animal research and were approved by the committee for humane treatment of animals at Shanghai Jiao Tong University School of Medicine.

Note that full information on the approval of the study protocol must also be provided in the manuscript.

Human research participants

Policy information about studies involving human research participants

Population characteristics	BM samples were obtained randomly from healthy donors and patients with CML (from 2011-2019) at diagnosis or at drug resistance. They are between 52 and 73 years old. The details of patients were provided in Supplementary Table 1.

Recruitment	Residual diagnostic specimens from CML patients at different clinical stages or at drug resistance were recruited. The selection of patients was random and we believe there was no bias in patients sample collection.
Ethics oversight	Informed consent was properly acquired at Shanghai Jiao Tong University School of Medicine affiliated Ruijin Hospital, Xinhua Hospital, Shanghai First People's Hospital and Shanghai Tongren Hospital.

Note that full information on the approval of the study protocol must also be provided in the manuscript.

Flow Cytometry

Plots

Confirm that:

- [x] The axis labels state the marker and fluorochrome used (e.g. CD4-FITC).
- [x] The axis scales are clearly visible. Include numbers along axes only for bottom left plot of group (a 'group' is an analysis of identical markers).
- [x] All plots are contour plots with outliers or pseudocolor plots.
- [x] A numerical value for number of cells or percentage (with statistics) is provided.

Methodology

Sample preparation	Mouse bone marrow was harvested by flushing two femurs and two tibias. Cells were re-suspended in FACS buffer (PBS with 0.1% BSA, 2mM EDTA) and incubated with indicated dyes for 30 minutes at 4°C in the dark, followed by washing with FACS buffer twice.
Instrument	Flow cytometry analysis was performed using LSRII (BD Biosciences).
Software	Data were analyzed with the FlowJo10 software package (FlowJo,LLC).
Cell population abundance	Flow cytometry analysis was performed on CD34/CD45 postive cell population after magnetic cell isolation.
Gating strategy	FSC-A/SSC-A for mononuclear cells, FSC-H/FSC-W followed by SSC-H/SSC-W for singlets. Cells were gated based on GFP or CD45, positive and negative markers.

- [x] Tick this box to confirm that a figure exemplifying the gating strategy is provided in the Supplementary Information.
