# Supplementary material for: Targeting USP47 overcomes tyrosine kinase inhibitor resistance and eradicates leukemia stem/progenitor cells in chronic myelogenous leukemia
Source: Nat Commun. 2021 Jan 4;12:51. doi: 10.1038/s41467-020-20259-0 (PMC7782553; doi:10.1038/s41467-020-20259-0)
Supplement: Supplementary file 2 — Peer Review File [file 41467_2020_20259_MOESM2_ESM.pdf]

Reviewers' comments:

Reviewer #1 (Remarks to the Author); expert on ubiquitin system:

Lei et al. present a manuscript where USP47 is shown to promote CML tumorigenesis by controlling YB-1-mediated DNA damage response. They found that USP47 protein is highly expressed in CML primary cells, physically binds to YB-1 and promotes its de-ubiquitination and stabilization. They further suggest using USP47 inhibitors to suppress the growth of either BCR-ABL-dependent or independent and insensitive to Imatinib CML cells, and present this both in vitro and in vivo. The manuscript is well-written and experiments are solid. I still have a few concerns about the conclusions made from the obtained results.

Major comments:

- What was the initial reason to study DUBs in CML tumors in particular in the first place? This is not clearly motivated in the introduction section.

- The effect of USP47 on the protein stability of YB-1 by CHX assay (figure 5e) should be addressed also after genetic and pharmacological inhibition of USP47 and/or in USP47 WT vs USP47 knockdown cells. Further, if YB-1 protein levels are regulated by ubiquitination/de-ubiquitination events why its levels are not affected by MG132?

- YB-1-mu shown in supplementary Figure 5c, should be described in the text.

- Figure 6j: the effect of USP47 knockdown on YB-1 protein levels should be investigated in USP47-/- MEFs after reintroduction of USP47.

- The role of USP47 on the de-ubiquitination of YB-1 reserves a deeper investigation. To prove the specificity of USP47, ubiquitination experiments shown in figure 5d should be performed also using YB-1 S2 mutant unable to bind USP47. More important, ubiquitination levels of YB-1 should be investigated in BM normal cells versus CML cells.

- In figure 7g, more than p53 levels, the authors should show phosphorylation levels of p53.

- Given that modulation of USP47 affects both YB-1 and Polβ, how the authors can assess that YB-1, and not Polβ, contributes to USP47-mediated DNA damage repair in CML cells? To address this point and in order to evaluate the biological role of USP47-mediated regulation of YB-1, the authors should study the effect of overexpression of USP47 in a context of YB-1 and/or Polβ depletion.

- βTrCP and YAP proteins should be mentioned between the substrates of USP47 in introduction.

- The dual role of USP47 as oncoprotein and tumor suppression should be included in the discussion.

- MW should be shown in all blots.

Reviewer #2 (Remarks to the Author); expert on DNA damage and CML:

In this work the authors identified BCR/ABL- ERK/STAT5 - USP47 - YB-1 pathway, which contributes to CML. While the finding is novel there are several problems with experimental design and interpretation of the data.
1) The postulation that USP47-YB-1 is required to repair DNA damage in CML cells is not

experimentally supported. g-H2AX observed after inhibition of USP47 might represent a secondary effect of cells already committed to apoptosis as detected by sub-G1 and active caspase3. Careful time-dependent analyses are required in addition to identification of DNA repair pathways affected by USP47. The data about the role of YB-1 in DNA repair in CML is also inadequate, showing only elevated levels of AP sites and decreased TOPO2a expression after inhibition of USP47.
2) It is confusing how AZD6738 (ATR inhibitor) can reduce the effect of USP47 inhibition on CML cells proliferation in the context of DNA damage?
3) The effect of USP47 inhibition on normal cells is not tested.
4) P22077 inhibits USP7 and USP47 deubiqitinases. Why the authors claim the effect via USP47? Also, P22077 inhibits the expression of USP47 protein, but not USP7 protein? The inhibitor should reduce enzymatic activities of USP7 and USP47, but not their expression?
5) Was there any toxicity in mice treated with P2207?
6) Primary CMLR cells should be tested in immunodeficient mice treated with P22077, followed by secondary bone marrow transplantations to examine the effect of the inhibitor on CML stem cells.
7) Figure 1a is hard to understand and interpret.

Reviewers' comments:

Reviewer #1 (Remarks to the Author); expert on ubiquitin system:

Lei et al. present a manuscript where USP47 is shown to promote CML tumorigenesis by controlling YB-1-mediated DNA damage response. They found that USP47 protein is highly expressed in CML primary cells, physically binds to YB-1 and promotes its de-ubiquitination and stabilization. They further suggest using USP47 inhibitors to suppress the growth of either BCR-ABL-dependent or independent and insensitive to Imatinib CML cells, and present this both in vitro and in vivo. The manuscript is well-written and experiments are solid. I still have a few concerns about the conclusions made from the obtained results.

Response: Thank you very much for your positive comments. We have conducted additional experiments to address your concerns, especially those related to the interaction between USP47 and YB-1.

Major comments:

1. What was the initial reason to study DUBs in CML tumors in particular in the first place? This is not clearly motivated in the introduction section.

Response: DUBs are emerging as novel therapeutic targets for a variety of cancers. In malignant hematological diseases, USP10, USP7, and USP2 have been shown to be involved in the pathogenesis of acute leukemia (Nat Chem Biol. 2017; 13, 1207–1215; Sig

Transduct Target Ther. 2018; 3, 29; Nat. Genet. 2015; 47: 330-337). Despite the fact that USP9X has been found to be involved in the survival of CML cells, little is known about the roles of other DUBs in the pathogenesis of CML. Therefore, using primary CML cells as a model, we examined the expression of DUBs. We have revised the introduction section of this paper and provided the initial reason for studying DUBs in CML.

2. The effect of USP47 on the protein stability of YB-1 by CHX assay (figure 5e) should be addressed also after genetic and pharmacological inhibition of USP47 and/or in USP47 WT vs USP47 knockdown cells. Further, if YB-1 protein levels are regulated by ubiquitination/de-ubiquitination events why its levels are not affected by MG132?

Response: According to your comments, we used USP47-specific shRNA to silence USP47 or USP47 inhibitor P22077 to treat K562 cells, and the half-life of YB-1 was examined by CHX assay (revised Figure 5i and Supplementary Figure 5b). Moreover, the half-life of YB-1 was also examined in MEF cells from wild type and Usp47-knockout mice (revised Figure 5j). The results showed that the stability of YB-1 could be controlled by USP47.

As for the effect of MG132 on protein level of YB-1, we have re-performed the experiment and confirmed that MG132 treatment led to the increase of YB-1 protein level (revised Figure 5k).

3. YB-1-mu shown in supplementary Figure 5c, should be described in the text.

Response: Thank you for pointing this out. YB-1-mu has been described in the text.

4. Figure 6j: the effect of USP47 knockdown on YB-1 protein levels should be investigated in USP47-/- MEFs after reintroduction of USP47.

Response: Thank you for the kind suggestion. As expected, the protein level of YB-1 is lower in Usp47-knockout than that in wild-type MEFs. The reintroduction of Usp47 in Usp47 knockout MEFs leads to the increase of YB-1 protein (revised Figure 5l).

5. The role of USP47 on the de-ubiquitination of YB-1 reserves a deeper investigation. To

prove the specificity of USP47, ubiquitination experiments shown in figure 5d should be performed also using YB-1 S2 mutant unable to bind USP47. More important, ubiquitination levels of YB-1 should be investigated in BM normal cells versus CML cells.

Response: Thank you for the brilliant suggestion. We co-transfected YB-1 full length/ S2 mutant/ S3 mutant and HA-Ub with or without USP47 into HEK293T cells, the results of which showed that USP47 cannot remove ubiquitin from YB-1 S2 truncation, indicating that the interaction between USP47 and YB-1 is important for USP47 to specifically deubiquitinates YB-1 (revised Figure 5d).

We also examined the ubiquitination levels of YB-1 in primary CML cells. The results showed that the ubiquitination level of YB-1 is lower in CML cells than that in normal bone marrow cells (revised Figure 5e).

6. In figure 7g, more than p53 levels, the authors should show phosphorylation levels of p53.

Response: Thank you for the suggestion. We have conducted additional experiments and found that the phosphorylation levels of p53 (Ser15) were not altered upon P22077 treatment (revised Figure 7f).

7. Given that modulation of USP47 affects both YB-1 and Polβ, how the authors can assess that YB-1, and not Polβ, contributes to USP47-mediated DNA damage repair in CML cells? To address this point and in order to evaluate the biological role of USP47-mediated regulation of YB-1, the authors should study the effect of overexpression of USP47 in a context of YB-1 and/or Polβ depletion.

Response: This is a good question. According to your suggestions, we silenced YB-1 or POLB in K562 and KBM5^{T315I} cells. We found that Polβ depletion cannot induce γH_2AX expression (revised Supplementary Fig. 6e), but YB-1 or USP47 knockdown did. We also overexpressed USP47 in YB-1 and/or Polβ depletion cells. The results demonstrated that overexpression of USP47 could not abrogate γH_2AX expression in YB-1 or YB-1/Polβ double silenced cells (revised Supplementary Fig. 6f), indicating that YB-1 contributes more to USP47-mediated DNA damage repair than Polβ in CML cells.

8. βTrCP and YAP proteins should be mentioned between the substrates of USP47 in introduction.

Response: Thank you for the suggestion. We have revised the text in the introduction.

9. The dual role of USP47 as oncoprotein and tumor suppression should be included in the discussion.

Response: Thank you for the suggestion. We have added this in the discussion.

10. MW should be shown in all blots.

Response: Thank you for the suggestion. We have revised the figures.

Reviewer #2 (Remarks to the Author); expert on DNA damage and CML:

In this work the authors identified BCR/ABL- ERK/STAT5 - USP47 - YB-1 pathway, which contributes to CML. While the finding is novel there are several problems with experimental design and interpretation of the data.

Response: Thank you very much for your positive comments. We have further designed some experiments to ensure the data are solid and revised some statements about data interpretation.

1) The postulation that USP47-YB-1 is required to repair DNA damage in CML cells is not experimentally supported. g-H2AX observed after inhibition of USP47 might represent a secondary effect of cells already committed to apoptosis as detected by sub-G1 and active caspase3. Careful time-dependent analyses are required in addition to identification of DNA repair pathways affected by USP47. The data about the role of YB-1 in DNA repair in CML is also inadequate, showing only elevated levels of AP sites and decreased TOPO2a expression after inhibition of USP47.

Response: To evaluate whether the γH_2AX observed after inhibition of USP47 is related to cell apoptosis, USP47 shRNA retrovirus was transfected into K562 and KBM^{T315I} cells.

Then, cells were collected at different time points (24, 48, 72, 96 h). The results showed that, with the knockdown of USP47, the increase of γH_2AX appeared before the cleavage of PARP1, indicating that the DNA damage appeared before cell apoptosis (revised Figure 6f and Supplementary Figure 6a).

To provide more evidence that YB-1 is involved in DNA damage repair in CML, we examined the protein levels of phosphorylated ATM, ATR, and PAR in YB-1-silenced K562 and KBM5^{T315I} cells in addication to γH_2AX expression. Interestingly, YB-1 knockdown increased the protein level of phosphorylated ATR and PAR (revised Figure 6h and Supplementary Fig. 6c), indicating an evoke of DNA damage repair response. On the contrary, overexpression of YB-1 in *Usp47*$^{-/-}$ MEFs could significantly rescue Usp47 knockout-induced γH_2AX expression (revised Figure 6l). All these data support that YB-1 contributes to USP47 inhibition-induced DNA damage response.

2) It is confusing how AZD6738 (ATR inhibitor) can reduce the effect of USP47 inhibition on CML cells proliferation in the context of DNA damage?

Response: Thank you for your professional opinion. This is actually related to the concentration of the inhibitor used, and we have described the concentration in the text. As USP47 inhibition leads to DNA damage and reduces cell proliferation, we speculate that blocking the DNA damage response by AZD6738 may partially rescue this effect. Indeed, AZD6738 at a lower concentration (50 nM) could reduce the cell viability inhibition of USP47 knockdown in CML cells. However, when used at higher concentration (2500 nM), the addition of AZD6738 could enhance the effect of USP47 knockdown on the proliferation of CML cells (revised Supplementary Fig. 6g).

3) The effect of USP47 inhibition on normal cells is not tested.

Response: The effect of USP47 inhibition by P22077 on PBMCs and CD34$^+$ cells has been done in Figure 7a and 7j. In the revised Figure 2d, we further demonstrated that knockdown of USP47 by shRNA does not affect the cell viability and colony formation activity of normal human CD34$^+$ cells.

4) P22077 inhibits USP7 and USP47 deubiqitinases. Why the authors claim the effect via USP47? Also, P22077 inhibits the expression of USP47 protein, but not USP7 protein? The inhibitor should reduce enzymatic activities of USP7 and USP47, but not their expression?

Response: In fact, we have addressed these questions in the discussion section of the original paper. "It has been reported that P22077 also inhibits the activity of USP7 and USP10. To find out the specific USPs that P22077 targets in CML cells, we measured the expression of substrates of USP7 and USP10, including p53, a substrate of both USP7 and USP10, and SIRT6, a substrate of USP10. As expected, P22077 increases the expression of p53 but has no effect on SIRT6 expression, indicating that P22077 has no obvious inhibitory effect on USP10 activity (revised Figure. 7f). Also, the knockdown of USP10 or USP7 does not affect the expression of YB-1 (revised Supplementary Figure. 7d). Moreover, P22077 still strongly inhibits cell viability in USP7 or USP10 stably-knockdown K562 cells (revised Supplementary Fig. 7e). Therefore, we deem that USP47 is the main effector for P22077 combating CML. Further investigations are warranted to develop a novel USP47-specific inhibitor for CML treatment."

For the effect on P22077 on the protein level of USP47, USP7, our explanation was "Interestingly, in addition to inhibit the activity of USP47, P22077 considerably reduces the protein level of USP47 in CML cells. P22077 may promote the degradation of USP47 by inhibiting the auto-deubiquitinating process of USP47, which is supported by the finding that MG132 reverses P22077-induced USP47 degradation (revised Supplementary Figure 7c)." Also, it is reported that USP47 deubiquitinates itself (Molecular and Cellular Biology. 2015 Sep; 35 (19) 3301-3311).

5) Was there any toxicity in mice treated with P22077?

Response: In our study, mice treated with P22077 (30 mg/kg) on a daily schedule for 2 weeks did not show obvious health problems or weight loss. Similar observation was obtained in other studies (Nat Med. 2016 Oct;22(10):1180-1186; Cell Death Dis. 2013 Oct; 4(10): e867).

6) Primary CMLR cells should be tested in immunodeficient mice treated with P22077, followed by secondary bone marrow transplantations to examine the effect of the inhibitor on CML stem cells.

Response: According to your suggestion, we tried to establish CML PDX model. Cells from TKI-resistant patients were collected and inoculated into B-NDG mice. Among the 6 patient-derived cells, one obtained success at last. Using this model, we found that P22077 treatment could significantly reduce the percentages of CML stem cells ($CD34^{+}CD38^{-}CD45^{+}$ cells) in the BM of mice after the secondary BM transplantation (revised Figure 7h, i). This result suggests that P22077 could reduce CML stem cells in PDX model.

7) Figure 1a is hard to understand and interpret.

Response: In Figure 1a, to investigate the possible DUBs involved in the pathogenesis of CML, we compared the mRNA levels of 84 DUBs in CML $CD34^{+}$ cells to those in normal $CD34^{+}$ cells. The most significantly upregulated USP47 was chosen for further investigation. To make it clear, we changed the label for Y-axis and revised the figure legend.

Taken together, we believe that the quality of the revised version has been greatly improved, and we look forward to your positive decision.

With best regards

Ying-Li Wu, M.D., Ph.D.

Hongqiao International Institute of Medicine, Shanghai Tongren Hospital/Faculty of Basic Medicine, Key Laboratory of Cell Differentiation and Apoptosis of National Ministry of Education, Shanghai Jiao Tong University School of Medicine, Shanghai 200025, China. Phone: 0086-21-63846590-776916; Fax: 0086-21-64154900, E-mail: wuyingli@shsmu.edu.cn

REVIEWERS' COMMENTS

Reviewer #1 (Remarks to the Author):

The authors have addressed all my questions and concerns. The manuscript is much improved and suitable for publication.

Reviewer #2 (Remarks to the Author):

The Authors addressed my concerns. I have a minor comment regarding point 5: The statement that P22077 did not show health problems should be included in the text.

We have followed the suggestions from the reviewers and revised our manuscript again. The point-by-point responses to reviewers' comments are as follows with red words for comments and black words for our response:

Reviewers' comments:

Reviewer #1 (Remarks to the Author):

The authors have addressed all my questions and concerns. The manuscript is much improved and suitable for publication.

Response: Thanks, we are happy to address your questions and concerns.

Reviewer #2 (Remarks to the Author):

The Authors addressed my concerns. I have a minor comment regarding point 5: The statement that P22077 did not show health problems should be included in the text.

Response: Thank you for your comment. We have included this statement in the text.

With best regards

Ying-Li Wu, M.D., Ph.D.

Hongqiao International Institute of Medicine, Shanghai Tongren Hospital/Faculty of Basic Medicine, Key Laboratory of Cell Differentiation and Apoptosis of National Ministry of Education, Shanghai Jiao Tong University School of Medicine, Shanghai 200025, China. Phone: 0086-21-63846590-776916; Fax: 0086-21-64154900, E-mail: wuyingli@shsmu.edu.cn
